# Alternative Splicing of Arabidopsis IBR5 Pre-mRNA Generates Two IBR5 Isoforms with Distinct and Overlapping Functions

**DOI:** 10.1371/journal.pone.0102301

**Published:** 2014-08-21

**Authors:** Thilanka Jayaweera, Chamindika Siriwardana, Sunethra Dharmasiri, Marcel Quint, William M. Gray, Nihal Dharmasiri

**Affiliations:** 1 Department of Biology, Texas State University, San Marcos, Texas, United States of America; 2 Department of Botany and Microbiology, University of Oklahoma, Norman, Oklahoma, United States of America; 3 Department of Plant Biology, University of Minnesota, St. Paul, Minnesota, United States of America; 4 Department of Molecular Signal Processing, Leibniz Institute of Plant Biochemistry, Halle (Saale), Germany; Umeå Plant Science Centre, Sweden

## Abstract

The *INDOLE-3-BUTYRIC ACID RESPONSE5* (*IBR5*) gene encodes a dual specificity phosphatase that regulates plant auxin responses. *IBR5* has been predicted to generate two transcripts through alternative splicing, but alternative splicing of *IBR5* has not been confirmed experimentally. The previously characterized *ibr5-1* null mutant exhibits many auxin related defects such as auxin insensitive primary root growth, defective vascular development, short stature and reduced lateral root development. However, whether all these defects are caused by the lack of phosphatase activity is not clear. Here we describe two new auxin insensitive *IBR5* alleles, *ibr5-4*, a catalytic site mutant, and *ibr5-5*, a splice site mutant. Characterization of these new mutants indicates that *IBR5* is post-transcriptionally regulated to generate two transcripts, *AT2G04550.1* and *AT2G04550.3*, and consequently two IBR5 isoforms, IBR5.1 and IBR5.3. The IBR5.1 isoform exhibits phosphatase catalytic activity that is required for both proper degradation of Aux/IAA proteins and auxin-induced gene expression. These two processes are independently regulated by IBR5.1. Comparison of new mutant alleles with *ibr5-1* indicates that all three mutant alleles share many phenotypes. However, each allele also confers distinct defects implicating IBR5 isoform specific functions. Some of these functions are independent of IBR5.1 catalytic activity. Additionally, analysis of these new mutant alleles suggests that *IBR5* may link ABP1 and SCF^TIR1/AFBs^ auxin signaling pathways.

## Introduction

The plant hormone auxin is a major regulator of plant growth and development. Auxin rapidly modulates gene expression through the degradation of Aux/IAA repressor proteins (Aux/IAAs). These repressor proteins interact with Auxin Response Factors (ARFs), which activate gene transcription. Auxin promotes the interaction between Aux/IAAs and SCF^TIR1/AFBs^ and thereby enhances the ubiquitination and degradation of Aux/IAA repressors through the 26S proteasome [1]. The degradation of Aux/IAAs relieves the repression on ARFs, leading to the modulation of gene transcription [2]. In this process, auxin interacts with its co-receptors TIR1/AFBs and Aux/IAAs, sequestering Aux/IAAs to the SCF^TIR1/AFBs^ protein complex [3], [4]. Therefore, the suppression of auxin responsive gene transcription in many auxin signaling mutants is due to reduced levels of Aux/IAA degradation [1], [2], [5]. The Arabidopsis *ibr5-1* was first identified in a genetic screen for mutants exhibiting resistant primary root growth to indole-3-butyric acid, a precursor of natural auxin indole-3-acetic acid (IAA) [6]. Subsequent analysis revealed that the *ibr5-1* mutant is also less sensitive to other natural and synthetic auxins and exhibits reduced auxin-induced gene expression. Interestingly, unlike in most other auxin insensitive mutants, Aux/IAA proteins are not stabilized [7], but rather degrade faster, in *ibr5-1* compared to the wild type suggesting that IBR5 negatively regulates the SCF^TIR1/AFBs^ pathway. Quite similar to *ibr5-1*, loss of Auxin Binding Protein1 (ABP1) function also enhances Aux/IAA degradation indicating that ABP1 negatively regulates the SCF^TIR1/AFBs^ pathway [8]. *IBR5* encodes one of the five (AtMKP1, AtMKP2, DsPTP1, PHS1 and IBR5) Arabidopsis dual specificity phosphatases that are involved in mitogen activated protein kinase (MAPK) pathways [9]. Therefore, identification of *ibr5-1* may link auxin signaling to MAPK pathways [9]. The Arabidopsis genome encodes 20 different MAPK proteins [10]. Of these, IBR5 physically interacts with MPK12 and de-phosphorylates the activated MPK12 [9].

Alternative splicing (AS) of genes to generate many transcripts, and thereby multiple protein isoforms, is a common mechanism found in eukaryotes. In plants, AS of genes has been implicated in growth, development and responses to environmental cues [11]. *IBR5* has also been predicted to generate two transcripts, *AT2G04550.1* and *AT2G04550.3*, with the possibility of producing two IBR5 isoforms, IBR5.1 and IBR5.3 (http://www.arabidopsis.org). However, previous work on *IBR5* has only identified a single transcript (*AT2G04550.1*) [6], [7]. While the previously isolated *ibr5-1* null-mutant exhibits many defective phenotypes [6], whether all of them are related only to the loss of *AT2G04550.1* is not clear.

*ibr5-1* also exhibits defects in ABA signaling [6], and ABA has been implicated in stress responses [12]. The IBR5 interacting protein, MPK12 plays a role in reactive oxygen species (ROS) mediated ABA signaling in guard cells [13]. Along with MPK9, MPK12 may also contribute to biotic stress tolerance [14]. In a recent study using yeast two hybrid assays, OsIBR5 was found to interact with tobacco MAP kinases, wounding induced protein kinase (WIPK), a homolog of OsMPK3, and salicylic acid induced protein kinase (SIPK), a homolog of OsMPK6 [15]. Moreover, over-expression of OsIBR5 in tobacco increases the sensitivity of transgenic plants to drought stress [9]. Therefore, emerging evidences suggest that IBR5 is involved in plant stress responses.

To dissect the role of IBR5 in plant hormone signaling and stress responses, we examined two additional mutant alleles of *IBR5*, *ibr5-4* and *ibr5-5*. While *ibr5-4* was isolated from a genetic screen for Arabidopsis mutants that were less sensitive to the synthetic auxin analog picloram, *ibr5-5* was isolated as an enhancer of *tir1-1* [16], [17]. Characterization of these new mutant alleles reveals that *IBR5* is post-transcriptionally regulated to generate two isoforms, IBR5.1 and IBR5.3, and IBR5.1 phosphatase activity is necessary for both proper Aux/IAA degradation and auxin-induced gene expression. Comparison of the three mutant alleles suggests that IBR5.1 and IBR5.3 isoforms may have distinct as well as overlapping functions in growth and development, and *IBR5* may mechanistically connect the ABP1 and SCF^TIR1/AFBs^ pathways.

## Results

### *ibr5-4* and *ibr5-5* are two new *ibr5* alleles

**Figure 1:**
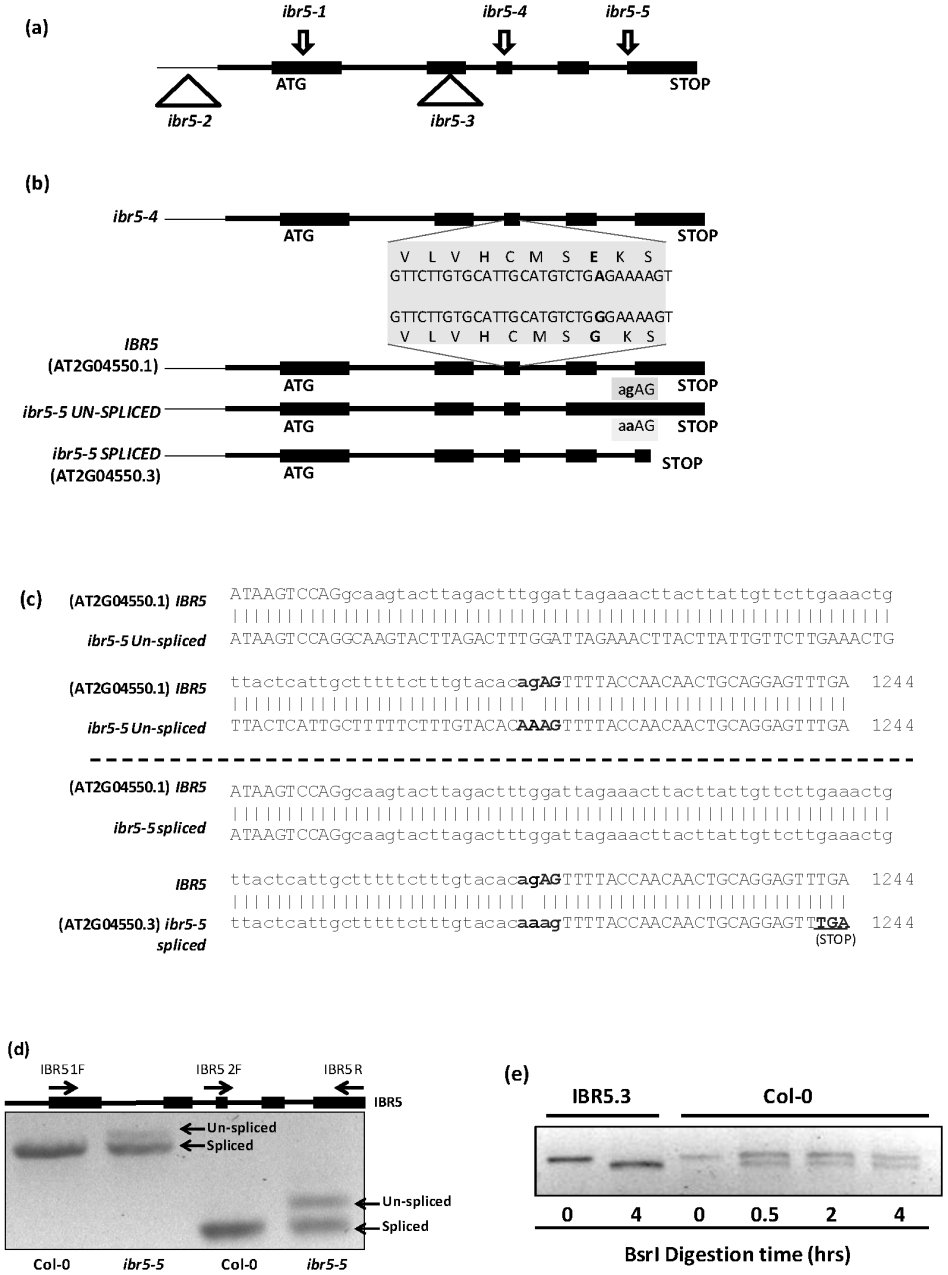
New mutant alleles of *IBR5*. a). Characterized alleles of *IBR5*. Arrows indicate the point mutations identified in *IBR5*. Triangles indicate the T-DNA insertions that alter or disrupt *IBR5* expression. b) *ibr5-4* has a G^727^ to A mutation in the third exon that changes G^132^ to E in the conserved dual-specificity phosphatase catalytic domain. *ibr5-5* has a G to A mutation in the intron of the last intron-exon junction. c) *IBR5* produces two mRNAs, one similar to the predicted splice variant *AT2G04550.3* and *AT2G04550.1* containing the last intron. d) mRNAs produced by *ibr5-5*. Total RNA was isolated from four day old *ibr5-5* and wild type Col-0 seedlings and cDNA was synthesized. *IBR5* was amplified using two different primer combinations (Table 1). e) Presence of IBR5.3 in wild type Col-0. *IBR5* was amplified from cDNA synthesized from Col-0 seedlings as described in (d). PCR products were subjected to *Bsr*I digestion up to four hours for complete digestion. *IBR5.3* amplified from *IBR5.3* clone was used as the positive control.

The Arabidopsis null mutant *ibr5-1* was previously identified through a genetic screen using indole butyric acid (IBA) [6]. To uncover additional genes involved in auxin response, we carried out a genetic screen using ethyl methanesulfonate-mutagenized Arabidopsis (Col-0) seeds to identify mutants that were resistant to the inhibitory effects of the synthetic auxin analog picloram. *ibr5-4* was isolated as a mutant that is moderately resistant to picloram, and the mutation was localized to *IBR5* by map-based positional cloning. The *ibr5-4* mutant has a G to A substitution at the 727^th^ position in the gene sequence, substituting the conserved Gly(G^132^) residue to Glu(E) in the highly conserved VxVHCxGxSRSxAYLM dual specificity phosphatase catalytic site (Figure 1a and b). The other allele, *ibr5-5*, was isolated as an enhancer of *tir1-1* [16], [17]. Map-based positional cloning and sequencing of *ibr5-5* revealed a G to A substitution at the 1216^th^ position located in the last intron-exon junction (Figure 1a,b and c). The mutation abolishes proper splicing of the 4^th^ intron of the *IBR5* pre-mRNA, and results in a longer transcript. Additionally, it also produces a shorter alternatively spliced transcript (Figure 1b–d). The unspliced mRNA in *ibr5-5* contains the last intron but shares the same stop codon as wild type *IBR5* (Figure 1b, 1c-upper panel and 1d). Therefore, if translated, it introduces 27 extra amino acids to the IBR5 protein (Figure S1). The alternatively-spliced mRNA in *ibr5-5* introduces a premature stop codon at position 1242 (Figure 1b, 1c-lower panel and 1d). Interestingly, this mRNA is similar to the predicted splice variant, *AT2G04550.3* (http://www.arabidopsis.org), in wild-type plants that is predicted to encode a truncated version of IBR5 (Figure S1). Similar to *ibr5-1* [6], both *ibr5-4* and *ibr5-5* are recessive alleles.

### *IBR5* is post-transcriptionally regulated

The presence of the predicted alternatively spliced mRNA variant in *ibr5-5* prompted us to test if the wild type Col-0 plants also produce two *IBR5* transcripts. If two transcripts are produced in wild type, they only differ by two bases making it difficult to distinguish them by size. However, closer examination of the two predicted mRNA sequences revealed that the alternatively spliced transcript (*AT2G04550.3*) contains a unique *Bsr*I restriction site. To test whether wild type *IBR5* is alternatively spliced, we prepared cDNA using mRNA isolated from 4-day old wild type Col-0 Arabidopsis seedlings. When *IBR5* was PCR-amplified using cDNA and digested with *Bsr*I, we detected two bands indicating the presence of both *AT2G04550.1* and *AT2G04550.3* transcripts (Figure 1e). To further confirm the presence of two *IBR5* transcripts, we cloned and sequenced both cDNAs from wild type as well as from *ibr5-5* (Figure S1). The two predicted peptide sequences of *AT2G04550.1* and *AT2G04550.3* transcripts are hereafter described as IBR5.1 and IBR5.3.

### Catalytic site mutation in *ibr5-4* affects IBR5.1 phosphatase activity

**Figure 2:**
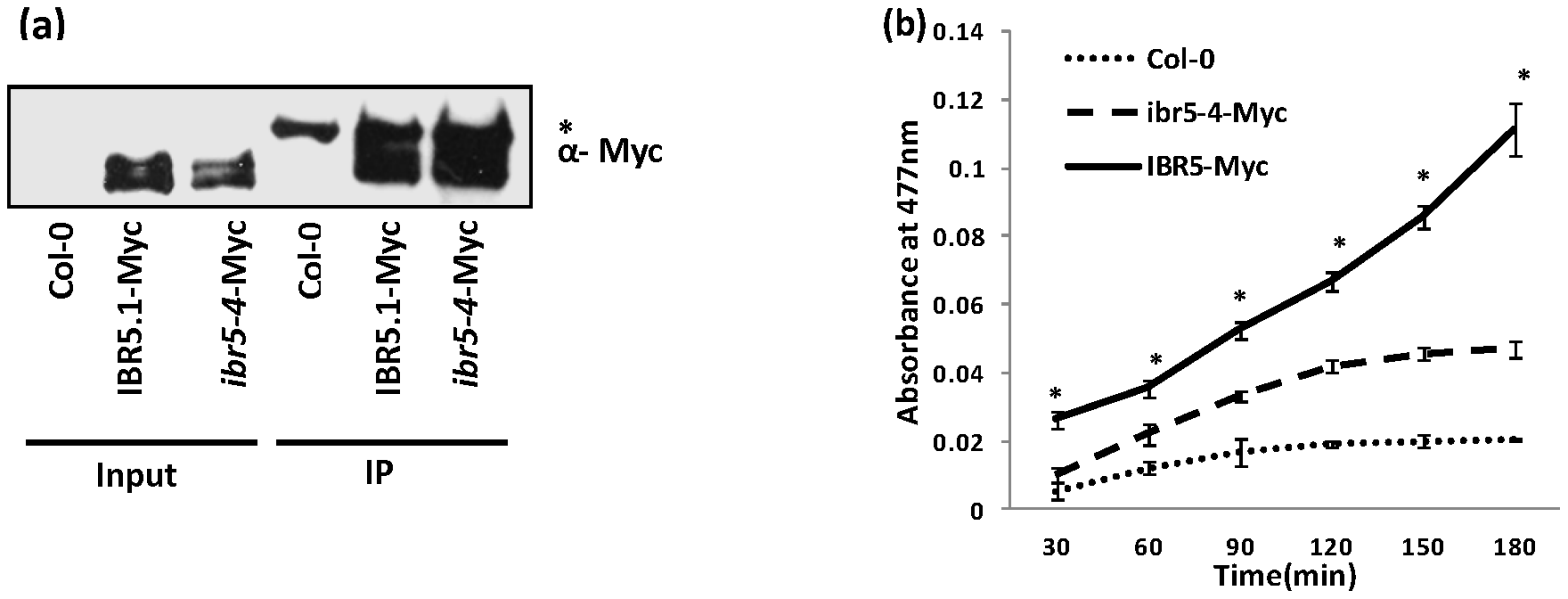
Catalytic activity of IBR5.1-Myc protein. a) Immuno-precipitation of IBR5.1-Myc and ibr5-4-Myc proteins. Total protein was isolated from transgenic plants over-expressing IBR5.1-Myc and ibr5-4-Myc. Tagged proteins were immuno-precipitated using anti-Myc antibody. 10% of the immuno-precipitate was visualized by western blotting using anti-Myc antibody. “*” indicates a non-specific protein that immuno-precipitates with anti-Myc antibody. b) The effect of *ibr5-4* mutation on IBR5 activity was measured using OMFP assay. Similar amounts of immuno-precipitates were used for the OMFP assay. Reactions were carried out in triplicate. Error bars indicate standard deviations from the means.

Our attempts to test phosphatase activity using bacterially expressed IBR5.1 or IBR5.3 failed due to the lack of enzyme activity in *E.coli* expressed protein. Therefore, to test whether the catalytic domain mutation in *ibr5-4* affects the enzyme activity, the IBR5.1, ibr5-4, and IBR5.3 proteins were stably expressed in Arabidopsis as C-terminal Myc tagged proteins using the constitutive *CaMV35S* promoter. The tagged proteins were immuno-precipitated using Myc-agarose beads. A portion of each immuno-precipitate was used for comparison of protein expression levels by western blot (Figure 2a). Equal amounts of proteins from immunoprecipitates were used for phosphatase assays employing the spectrophotometric substrate, OMFP [9]. As shown in Figure 2b, *IBR5.1-Myc* immunoprecipitate exhibited higher phosphatase activity than control precipitate. However, this activity was drastically reduced in *ibr5-4-Myc* immunoprecipitate, indicating that the *ibr5-4* mutation affects phosphatase activity. Although we tested the phosphatase activity of IBR5.3 isoform using *IBR5.3-Myc* immunoprecipitate, we did not detect phosphatase activity (Figure S2).

### IBR5.1 catalytic activity is necessary for both Aux/IAA degradation and auxin induced gene expression

**Figure 3:**
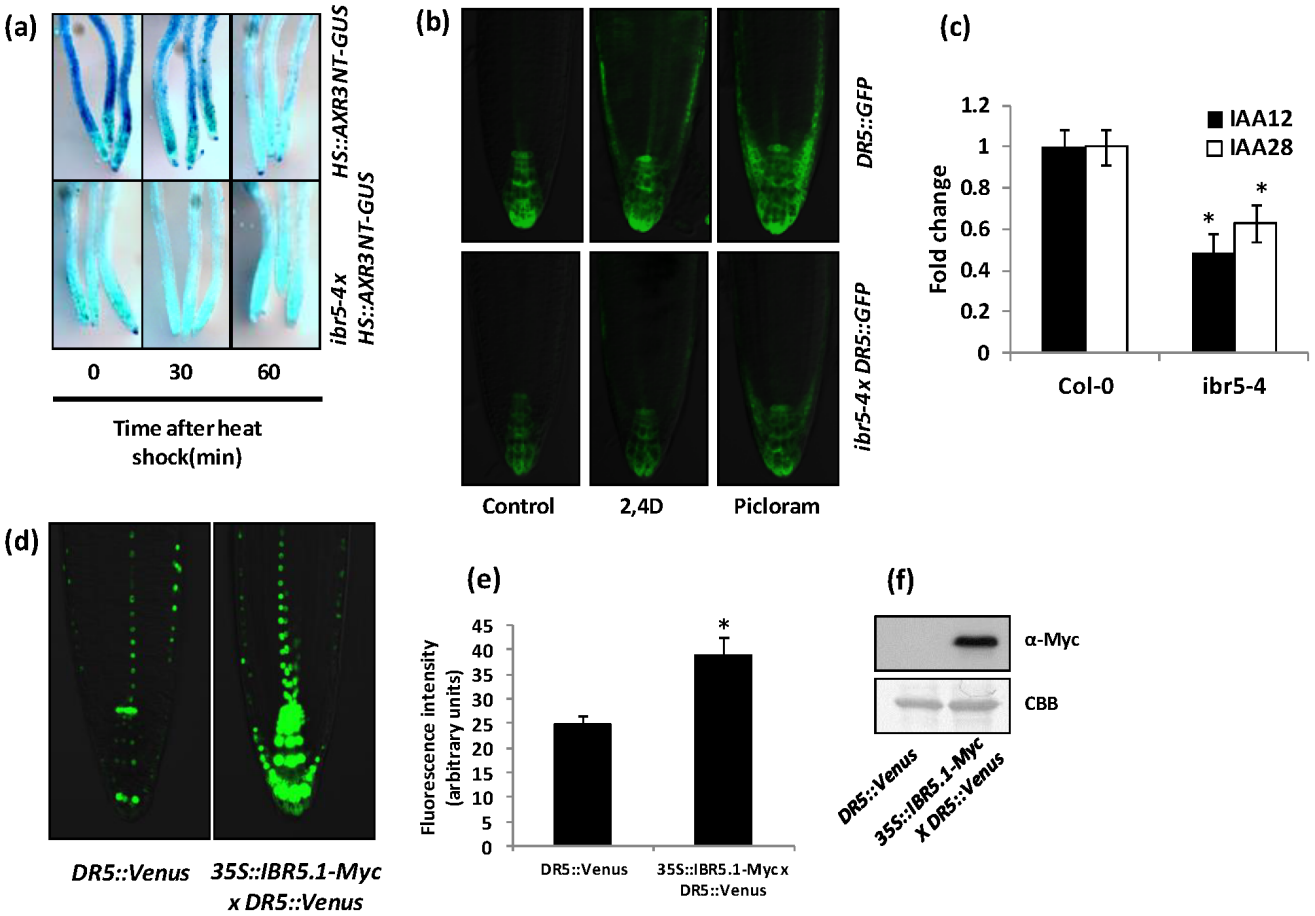
Aux/IAA degradation and auxin induced *DR5::GFP* expression. a) Rapid degradation of *AXR3NT-GUS* in *ibr5-4*. Four day old light grown wild type Col-0 and *ibr5-4* seedlings carrying *HS::AXR3NT-GUS* were heat shocked for two hours, fixed after the indicated time intervals and stained for GUS. b) Reduced *DR5::GFP* expression in *ibr5-4*. Four day old light grown wild type Col-0 or *ibr5-4* seedlings carrying the *DR5::GFP* auxin inducible reporter were used. Seedlings were treated with mock (ethanol/DMSO), 100 nM 2,4D or 10 µM picloram for 3 hrs and imaged using Olympus FV1000 confocal microscopy. c) Expression of *IAA12* and *IAA28* were assessed by qRT-PCR using 4 day old Col-0 and *ibr5-4* seedlings. UBA (*AT1G04850*) was used as the internal control. Expression levels were normalized against wild type Col-0. Error bars indicate standard deviations from the mean. Stars indicate that the means differ significantly from the respective control (ANOVA, P<0.05). d) Increased *DR5::Venus* expression in *IBR5.1-Myc* background. Four day old light grown wild type Col-0 or *IBR5.1-Myc* transgenic seedlings carrying *DR5::Venus* were used. Seedlings were imaged using Olympus FV1000 confocal microscopy. e) Quantitative analysis of Venus expression. Expression of Venus was quantified using ImageJ software. Error bars indicate standard deviation from the mean. Stars indicate that the means differ significantly from the control (n = 15, Student's t-test, P<0.05). f) Expression of *IBR5.1-Myc* in *DR5::Venus* lines. Total protein was isolated from homozygous seedlings carrying *IBR5.1-Myc* and *DR5::Venus*. *IBR5.1-Myc* was visualized by western blotting using anti-Myc antibody.

Unlike in many other auxin insensitive mutants, *AXR3NT-GUS* reporter protein is not stabilized in *ibr5-1* compared to wild type [7]. However, *ibr5-1* is a null mutant lacking both IBR5 isoforms. Additionally, overexpression of IBR5.1 carrying a catalytic site mutation (*35S::IBR5^C129S^*) completely or partially rescued some *ibr5-1* phenotypes [7], raising the question of whether IBR5.1 catalytic activity is important for the regulation of Aux/IAA degradation. To address this issue, we crossed *ibr5-4* that carries a mutation in the catalytic domain, into the *HS::AXR3NT-GUS* line and selected lines homozygous for both *ibr5-4* and the transgene. When these lines were tested for GUS activity, similar to previous findings with *ibr*5*-1*, degradation of *AXR3NT-GUS* was not stabilized, but rather it was accelerated in *ibr5-4* compared to that of wild type (Figure 3a).

Auxin induced gene transcription is impaired in *ibr5-1*, as shown by the reduced induction of *DR5::GUS* reporter gene [6]. Nevertheless, loss of both IBR5 isoforms in *ibr5-1* null mutant again raises the question of whether IBR5.1 catalytic activity is necessary for auxin induced gene transcription. To test this possibility, we crossed the *ibr5-4* mutant into the *DR5::GFP* reporter line and obtained plants homozygous for both the mutation and the transgene. The expression of GFP was less in *ibr5-4* compared to wild type background. In addition, the induction of *DR5::GFP* by 2,4-D and picloram was also reduced in *ibr5-4* mutant background compared to the wild type (Figure 3b). To further analyze the change in auxin-induced gene expression in *ibr5-4*, we compared the expression of *Aux*/*IAA12* and *Aux*/*IAA28* transcripts in *ibr5-4* and Col-0 wild type backgrounds using qRT-PCR. Results showed that the expression of both *Aux/IAA* genes was down regulated in *ibr5-4* (Figure 3c). As the *ibr5-4* mutation reduced auxin induced gene expression, we hypothesized that over-expression of IBR5.1 might enhance auxin regulated gene expression. To test this hypothesis, we crossed the *35S::IBR5.1-Myc* over-expression line into the *DR5::Venus* reporter line [18]. When we tested lines homozygous for both transgenes, the expression of Venus was significantly higher in IBR5-Myc over-expression background compared to wild type (Figure 3d–f) suggesting that higher phosphatase activity of IBR5.1 enhances auxin regulated gene expression.

### IBR5.1 and IBR5.3 isoforms may have distinct and overlapping functions

**Figure 4:**
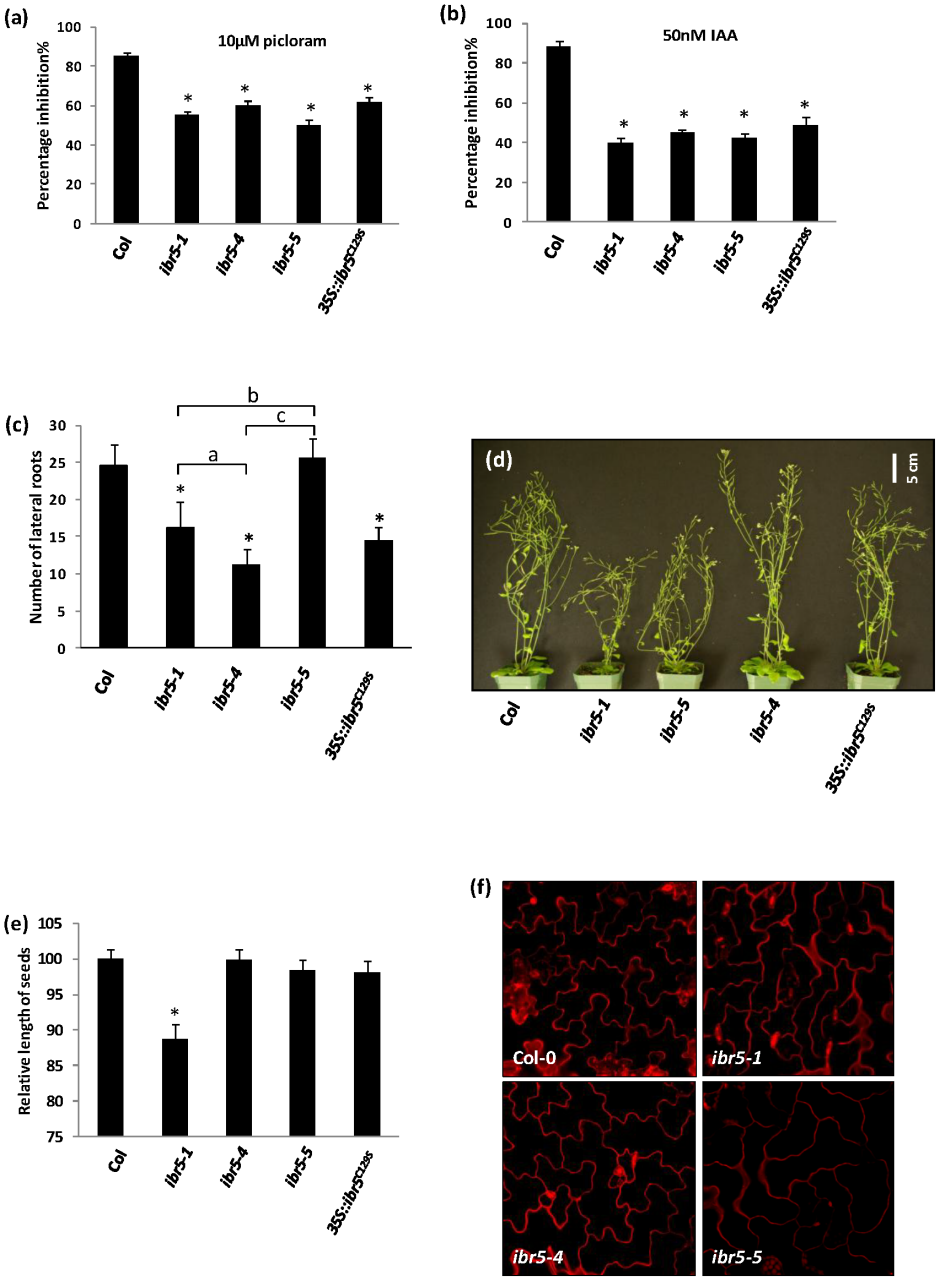
Characterization of new *ibr5* alleles. a–b) Inhibition of primary root elongation by auxin. Seedlings were grown on unsupplemented ATS media for four days and transferred onto ATS containing the indicated concentrations of a) picloram, b) IAA. Seedlings were grown for four additional days, and the length of the primary root was measured. Results were normalized against unsupplemented media. Error bars indicate standard error of the mean. Stars indicate the means that differ significantly from the control (n = 15). c) Number of lateral roots in *ibr5* mutants. Seedlings were grown on unsupplemented ATS media for 12 days, and the number of primordia emerged from the primary root were counted as lateral roots using a dissecting microscope. Error bars indicate standard deviations of the means (n = 20). d) Adult plant morphology of six week old *ibr5* alleles grown in continuous light at 25°C. e) Seed size of *ibr5* mutants. Dried mature seeds were photographed and the lengths of the seeds were measured using ImageJ software. Error bars indicate standard deviations of the means (n = 20). Stars indicate the means that differ significantly from the control; letters indicate the samples that differ significantly from each other (ANOVA, Tukey's HSD, P<0.05). f) Interdigitation of leaf epidermal pavement cells of *ibr5* alleles. Propidium iodide stained lower epidermis of seven-day old cotyledons were imaged.

Since there are two transcripts of *IBR5*, we hypothesized that the three mutant alleles may exhibit specific mutant phenotypes while some phenotypes may be shared. As predicted, some of the phenotypes such as incomplete vascular development (Figure S3), epinastic leaves, and serrated rosette and cauline leaves, shown by *ibr5-4* and *ibr5-5* mutants were similar to the null mutant *ibr5-1* [6], [7]. However, all of the above phenotypes were weaker in *ibr5-4* when compared to *ibr5-1* or *ibr5-5*. Similar to *ibr5-1*, light grown *ibr5-4* and *ibr5-5* mutant seedlings displayed shorter hypocotyls and longer roots compared to wild type Col-0 (Figure S4a, b). Nevertheless, the hypocotyls of *ibr5-4* and *ibr5-5* were slightly longer compared to *ibr5-1*. We also tested these two new mutant alleles for the inhibition of primary root elongation by auxins. Similar to *ibr5-1*, primary root elongation of both *ibr5-4* and *ibr5-5* mutants was less sensitive to picloram, IAA, 2,4-D and IBA (Figure 4a,b and S5a,b).

We also studied the differences among the three mutant alleles. In these experiments, we also included the previously published transgenic line, *35S::ibr5^C129S^*, which carries C129S catalytic site mutation in *ibr5-1* mutant background [7]. According to previous results, *ibr5-1* produces shorter and slightly fewer numbers of lateral roots compared to the wild type [6]. A reduced number of lateral roots was also observed in *ibr5-4* (Figure 4c), however the lateral root number of *ibr5-5* was similar to that of wild type suggesting that the presence of the splice variant (*At2G04550.3*) is sufficient for proper lateral root development (Figure 4c). There were also differences in plant height among the different alleles. At mature stages both *ibr5-5* and *ibr5-1* plants were shorter than the wild type, but *ibr5-4* mutants and *35S:IBR5^C129S^* had the same height as wild type plants ([7] and Figure 4d). Furthermore, *ibr5-1* seeds were smaller, but *ibr5-4*, *ibr5-5* and *35S:IBR5^C129S^* seeds were similar in size compared to wild type (Figure 4e).

Recent studies show that ABP1 and Rho GTPase dependent auxin signaling is involved in pavement cell interdigitation in Arabidopsis leaves [19]. Additionally, recent work indicates that ABP1 is a negative regulator of the SCF^TIR1/AFBs^ pathway [8]. These findings led us to examine the leaf epidermal cells of *ibr5* mutant alleles. Interestingly, in both *ibr5-1* and *ibr5-5*, interdigitation of leaf epidermal pavement cells is compromised compared to wild type. However, this phenotype is less obvious in both *ibr5-4* (Figure 4f) and *35S::IBR5^C129S^* (Figure S8).

**Figure 5:**
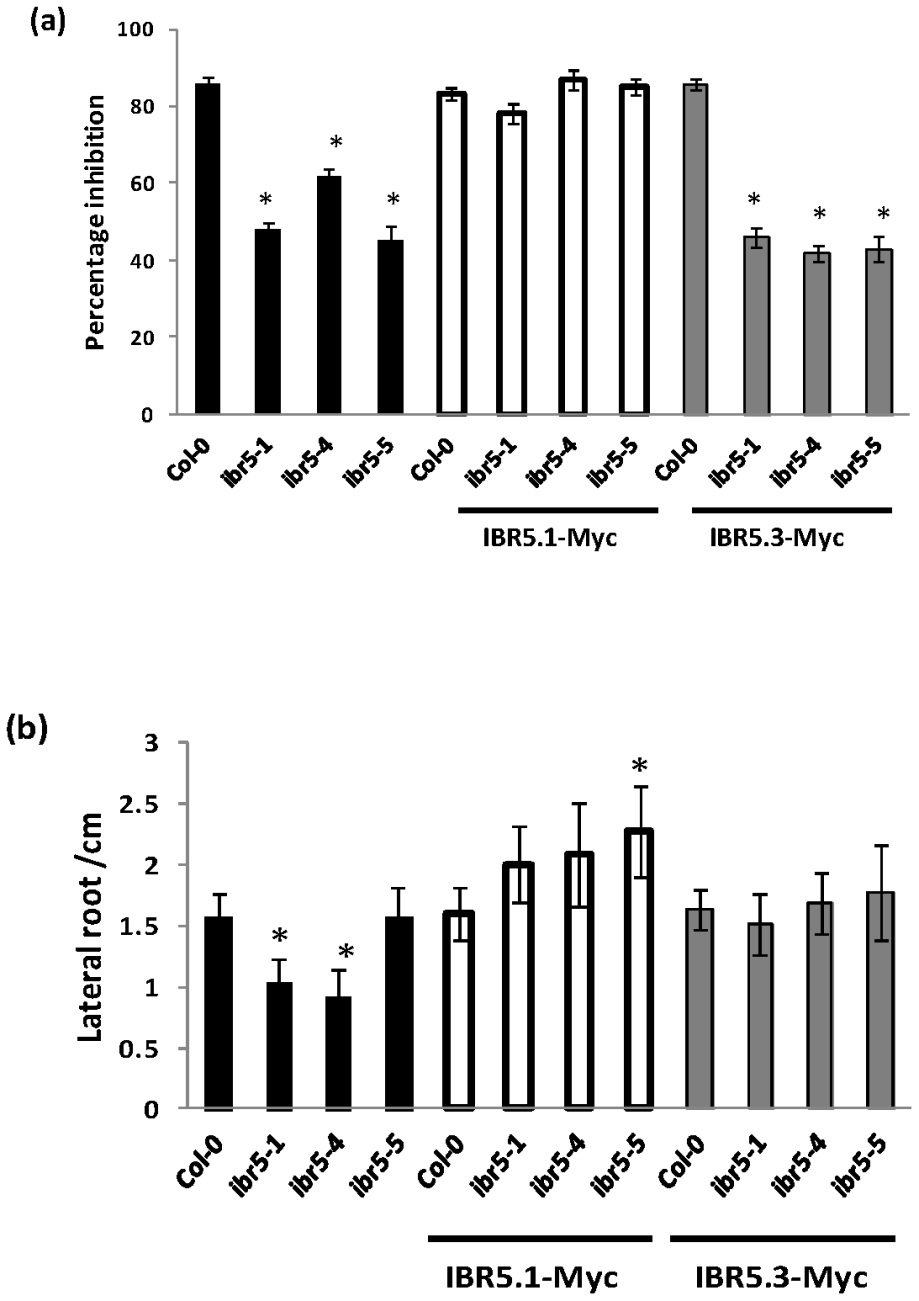
Complementation of *ibr5* mutants by overexpression of IBR5.1 and IBR5.3. a) Complementation of auxin resistant primary root elongation of *ibr5* mutants by *IBR5.1-Myc* but not *IBR5.3-Myc* over-expression. The root growth assay was performed as described in figure 4a. Error bars indicate standard error of the mean. b) Complementation of reduced lateral root phenotype of *ibr5* mutants by *IBR5.1-Myc* and *IBR5.3-Myc* overexpression. The experiment was performed as described in figure 4c. Error bars indicate standard deviations from the means. Stars indicate that the means differ significantly from the control (n = 20, ANOVA, P<0.05).

To understand the specific functions of IBR5.1 and IBR5.3 isoforms, we over-expressed IBR5.1 and IBR5.3 in Col-0, *ibr5-1*, *ibr5-4* and *ibr5-5* backgrounds. When we tested lines homozygous for the transgene, IBR5.1 but not IBR5.3 complemented the auxin insensitive primary root elongation phenotype (Figure 5a) and the interdigitation defect of leaf epidermal cells (Figure S8) of the three mutant alleles. However, both IBR5 isoforms complemented the defective lateral root phenotype of *ibr5-1* and *ibr5-4* (Figure 5b).

### IBR5 isoforms exhibit different localization patterns

**Figure 6:**
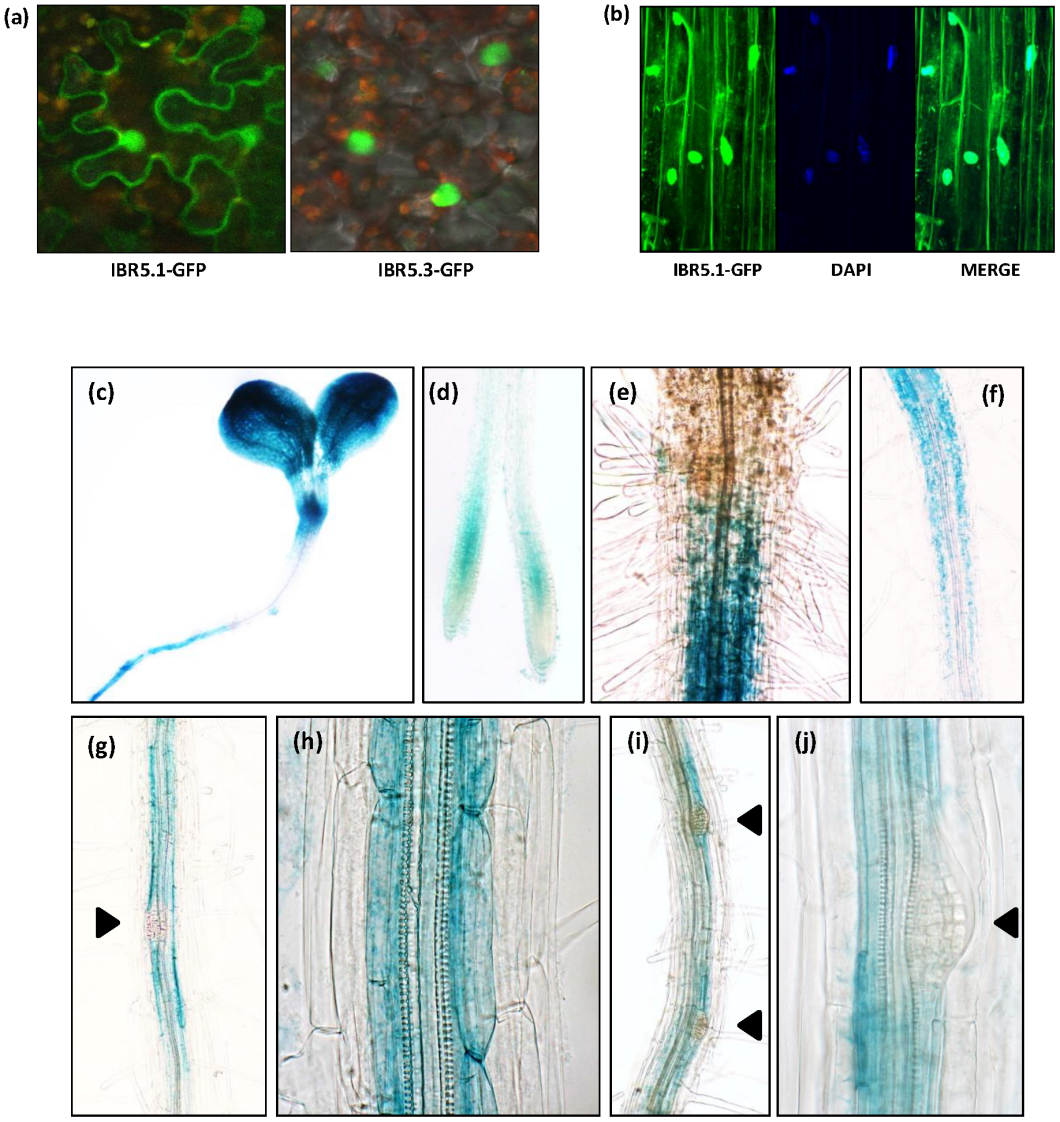
Subcellular localization and tissue specific expression of IBR5. a) Sub-cellular localization of IBR5.1 and IBR5.3 isoforms. *35S::IBR5.1-GFP* and *35S::IBR5.3-GFP* reporter constructs were transiently expressed in *Nicotiana benthamiana* leaves and images were acquired using Olympus FV1000 confocal microscopy. b) Expression of IBR5.1-GFP in Arabidopsis seedlings. The *IBR5::IBR5.1-GFP* translational reporter construct was stably expressed in Arabidopsis. Expression of *IBR5.1-GFP* in root cells was detected using Olympus FV1000 confocal microscopy. (c–j) Tissue specific expression of *IBR5.1-GUS* in Arabidopsis seedlings. The *IBR5::IBR5-GUS* translational reporter construct was used to examine tissue specific expression of IBR5. Seven day old light grown seedlings were fixed and stained for GUS. IBR5 expression in the (c) cotyledons and top region of the hypocotyl, (d) elongation zone of the root tip, (e) shoot-root junction, (f) pericycle and endodermis of the mature region of the root, (g–j) adjacent pericycle and endodermis cells of lateral root initiation sites.

To study the sub-cellular localization of IBR5.1 and IBR5.3, we generated *35S::IBR5.1-GFP* and *35S::IBR5.3-GFP* gene constructs and transiently expressed them in *Nicotiana benthamiana* leaves, and GFP fluorescence was observed using confocal microscopy. While *IBR5.1-GFP* is localized to the nucleus and the cytoplasm, *IBR5.3-GFP* is exclusively localized to the nucleus (Figure 6a and S6a, b). To further study the expression pattern and sub-cellular localization, *IBR5::IBR5.1-GFP* and *IBR5::IBR5.1-GUS* translational reporter constructs were stably expressed in Arabidopsis. These constructs can be used to evaluate *IBR5* promoter activity and the expression of the IBR5.1 isoform. Contrary to previous results [9], our results indicate that IBR5.1 localizes to the nucleus and the cytoplasm (Figure 6b). This is also consistent with the sub-cellular localization of OsIBR5 [15]. Expression of *IBR5::IBR5.1-GUS* in shoots of both light grown (Figure 6c) and dark grown (Figure S7) seedlings was restricted to the region surrounding shoot apical meristem and cotyledons. The expression pattern in remaining parts of the shoot was similar to that of previously observed results using the *IBR5::GUS* transcriptional fusion [6].

*IBR5::IBR5.1-GUS* was expressed in the pericycle and endodermis of the mature region of the root (Figure 6e–h). Correlating with the reduced number of lateral roots observed in *ibr5-1* and *ibr5-4*, the expression of the reporter gene was observed in the cells adjacent to xylem pole pericycle cells and in endodermis cells near the lateral root initiation sites. However, GUS expression was absent in dividing cells of the lateral root primordia (Figure 6g,i,j). Similarly, *IBR5* was expressed in the elongation zone but not in the division zone of the root tip (Figure 6d).

### IBR5 is involved in stress responses

**Figure 7:**
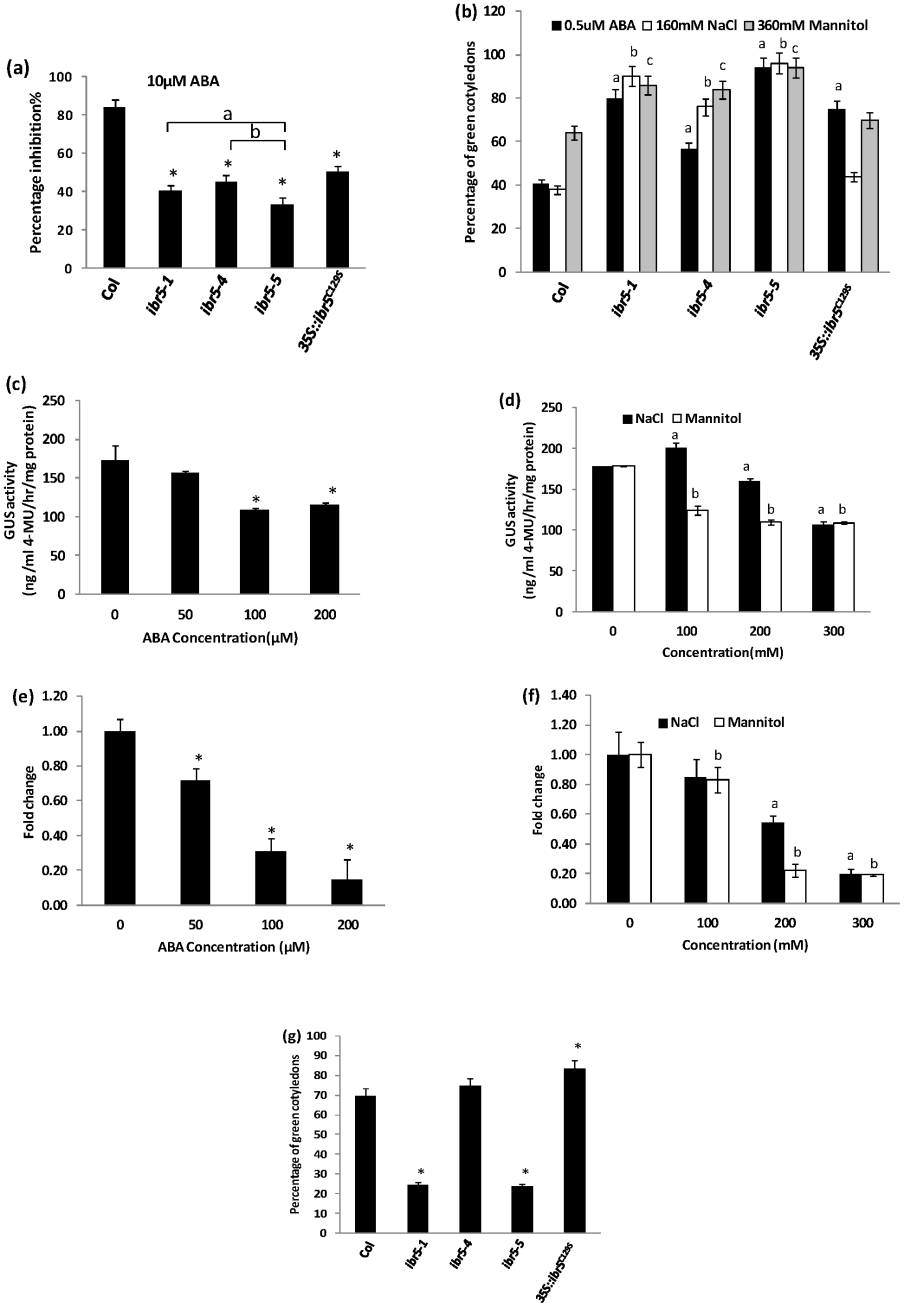
ABA and stress responses of *ibr5* alleles. a) inhibition of primary root elongation by ABA. Four day old seedlings were transferred on to ATS containing 10 µM ABA, and the length of the primary root was measured after 4 day incubation at 21°C under continuous illumination. Error bars indicate standard error of the mean (n = 15). Stars indicate that the means differ significantly from the control, letters indicate the samples that differ significantly from each other (ANOVA, Tukey's HSD, P<0.05). b) Post germination inhibition by ABA and stresses. Seeds stratified at 4°C for two days were grown on media containing indicated amounts of ABA, NaCl or mannitol. Seedlings with green cotyledons were counted after 7 days of growth, and percentage was calculated. c–d) Expression of *IBR5::IBR5.1-GUS* in response to ABA and stresses. Four day old *IBR5::IBR5.1-GUS* transgenic seedlings treated with various concentrations of ABA, NaCl or mannitol for 18 hrs were used to perform quantitative GUS assays. Each data point indicates the mean value of 3 replicates. e–f) Expression of IBR5 in response to ABA and stresses. Four day old Col-0 seedlings treated with various concentrations of ABA, NaCl and mannitol for 18 hrs were used. Expression of IBR5 was assessed by qRT-PCR. UBA (*AT1G04850*) was used as the internal control. g) Response of *ibr5* alleles to oxidative stress in post germination growth. The experiment was performed as described in (b) in the presence of 1 µM methyl viologen. Seedlings with green cotyledons were counted after 7 days of growth, and percentage was calculated. Error bars indicate standard deviation from the mean. Stars or letters indicate that the means differ significantly from the respective control (ANOVA, P<0.05).

As *ibr5-1* is defective in ABA responses [6], we tested *ibr5-4* and *ibr5-5* for root elongation and post-germination growth inhibition by ABA. All alleles were less sensitive to inhibition of primary root elongation (Figure 7a) and post-germination inhibition by exogenous ABA (Figure 7b). Similar to auxin, ABA also regulates gene transcription to modulate plant growth and development. Therefore, the effect of ABA on expression of *IBR5* was studied using the *IBR5::IBR5.1-GUS* reporter line. Prolonged ABA treatment down-regulated *IBR5* expression in a concentration dependent manner (Figure 7c). Similarly, qRT-PCR analysis showed that the expression of endogenous *IBR5* was suppressed by ABA (Figure 7e).

Since most of the previously reported ABA response mutants exhibit altered responses to abiotic stress [20], we tested all three *ibr5* alleles for salinity, osmotic and oxidative stresses using NaCl, mannitol and methyl viologen (MV) respectively. Even though the mutants did not differ significantly from wild type in inhibition of primary root elongation (Figure S9), all the alleles were resistant to post-germination inhibition when compared to wild type on NaCl and mannitol (Figure 7b). Nevertheless, the *35S::IBR5^C129S^* line was as sensitive as wild type to both salinity and osmotic stress, though it was resistant to ABA. Interestingly, as indicated by *IBR5::IBR5.1-GUS* expression, these stress conditions down regulated the expression of *IBR5.1-GUS* except a slight increase at low salt concentration (Figure 7d). To validate our results, we also tested the expression of endogenous *IBR5* under the same NaCl and mannitol concentrations using qRT-PCR. As expected, the *IBR5* expression was down regulated in response to salinity and osmotic stress (figure 7f). To study the effect of oxidative stress on *ibr5* mutants, we tested *ibr5* mutant lines for post germination inhibition on a low concentration, (1 µM) of MV. At this concentration both *ibr5-1* and *ibr5-5* were hypersensitive to MV while wild type, *ibr5-4* and *35S::IBR5^C129S^* lines were much less affected (Figure 7g).

## Discussion

Among the five Arabidopsis dual specificity phosphatases, IBR5 is involved in auxin signaling. The null allele *ibr5-1* shows auxin insensitive primary root growth and several other mutant phenotypes [6], [7]. Nevertheless, IBR5 phosphatase activity may not be necessary for its full spectrum of functions [7]. *IBR5* has been predicted to undergo alternative splicing to generate two transcripts, *AT2G04550.1* and *AT2G04550.3*, consequently producing IBR5.1 and IBR5.3 isoforms (http://www.arabidopsis.org). Therefore, the possibility of alternative splicing of *IBR5* may complicate observed phenotypes of *ibr5-1*. Here we describe two new mutant alleles of *IBR5* which give more insight into its functions that include functions independent of its catalytic activity and also possible functions of the previously unknown IBR5.3 isoform. *ibr5-4* is a catalytic site mutant in which Glycine (G) in the VxVHCxGxSRSxAYLM highly conserved catalytic site [21] is substituted with Glutamic acid (E) (Figure 1b). This G to E substitution disrupts the IBR5.1 catalytic activity (Figure 3b). Thus, the *ibr5-4* allele is a more reliable alternative to evaluate the requirement of phosphatase activity in the auxin signaling pathway.

### IBR5 is alternatively spliced

In this study we have conclusively demonstrated that *IBR5* generates two transcripts, *AT2G04550.1* and *AT2G04550.3*, suggesting that both IBR5.1 and IBR5.3 isoforms are present in Arabidopsis. In *ibr5-1*, there is a premature stop codon in the 1^st^ exon [6], suggesting that both IBR5 isoforms are absent. Since *ibr5-4* is a catalytic site substitution, both IBR5 isoforms should be mutated at the conserved catalytic site. In *ibr5-5*, the predicted IBR5.1 polypeptide has 27 extra amino acid residues due to the unspliced 4^th^ intron, but the IBR5.3 isoform is intact (Figure S1). The availability of three mutant alleles that affect the functions of two different IBR5 isoforms in different ways provides the opportunity to dissect *IBR5*'s functions in Arabidopsis growth and development. A previous study [9] along with our data (Figure 2b) confirms the phosphatase activity of IBR5.1 isoform. The predicted IBR5.3 isoform also contains the highly conserved catalytic site, but lacks the last few amino acids of the conserved dual specificity catalytic domain (Figure S1). We did not detect phosphatase activity using IBR5.3-Myc recombinant protein (Figure S2). The lack of phosphatase activity in IBR5.3-Myc could be either due to the loss of last 10 amino acids of the phosphatase catalytic domain or the proximity of the Myc tag to the catalytic site. Nevertheless, the fact that *ibr5-5* is resistant to both auxins and ABA also suggests that IBR5.3 lacks phosphatase activity.

Alternative splicing of pre-mRNA is a common regulatory process in both plants and animals to increase the diversity of proteins. According to recent RNA sequencing data, about 42 to 61% of the intron containing genes in Arabidopsis are alternatively spliced [22], [23]. Previous studies show that alternative splicing can be regulated by various stress conditions. For example, the circadian clock gene *CCA1* predominately produces the normal transcript under cold stress, but produces an alternative mRNA isoform with a premature termination codon under heat stress [24]. Similarly in Arabidopsis, nutrient deficiency [25], temperature shift [26], [27], and pathogenicity [28] have been shown to regulate alternative splicing suggesting that it may be required for plant adaptation to stress. Interestingly, a recent study has shown that *YUCCA4*, a gene involved in the auxin biosynthesis pathway, is alternatively spliced in tissue specific manner. *YUCCA4* produces two catalytically active isoforms YUCC4.1 and YUCCA4.2, which are differentially localized in the cell [29]. Similarly, alternative splicing of Arabidopsis *ZIFL1* results in two isoforms, ZIFL1.1 and ZIFL1.3, each with distinct sub-cellular localization and function [30]. Our study indicates that while the IBR5.1 isoform with phosphatase activity localizes to the cytosol and the nucleus, the IBR5.3 isoform, which doesn't show phosphatase activity, is exclusively localized to the nucleus (Figure 6a and S6). Genetic analysis of *ibr5* mutants indicates that lack of IBR5.1 phosphatase activity results in auxin and ABA insensitive primary root growth, and these defective phenotypes cannot be rescued by IBR5.3 suggesting that these two isoforms have distinct functions. Conversely, the presence of IBR5.3 is sufficient for normal lateral root development (Figure 4c and 5b), and defective lateral root development can be rescued by either IBR5.1 or IBR5.3 suggesting that these two isoforms may have some overlapping functions. It will be interesting to determine whether alternative splicing of *IBR5* is regulated by developmental or environmental cues as well as the biological significance of restrictive nuclear localization of IBR5.3.

### Some IBR5 functions are independent of IBR5.1 catalytic activity

The *ibr5-1* mutant has a short stature compared to wild type [6], [7]. Similarly, *ibr5-5* is also shorter than wild type but *ibr5-4* does not show any defect in plant height (Figure 4d) suggesting that IBR5.1 phosphatase activity may not be necessary for proper plant height. This is further supported by the ability of *35S::IBR5^C129S^* to rescue plant height in *ibr5-1* to resemble wild type ([7] and Figure 4d). On the other hand, the short plant phenotype in *ibr5-5* may be due to the absence of the IBR5.1 protein, or non-functional IBR5.1 protein with an aberrant C-terminus. *ibr5-1* also produces smaller seeds compared to wild type (Figure 4e). However, this phenotype is not apparent in either *ibr5-4*, *ibr5-5* or *35S:IBR5^C129S^* lines. Similarly, *ibr5-1* and *ibr5-5* are hypersensitive to MV while wild type, *ibr5-4* and *35S::IBR5^C129S^* lines are less affected (Figure 7g). Taken together, these results suggest that IBR5.1 catalytic activity may not be necessary for these processes. However, primary root elongation of all three *ibr5* alleles and *35S:IBR5^C129S^* is less sensitive to both auxins and ABA. Over-expression of IBR5.1-Myc, but not IBR5.3-Myc, complements the auxin insensitive primary root growth (Figure 5a). Taken together, these results suggest that catalytic activity of IBR5.1 is necessary for both auxin and ABA responses, but further genetic analysis may be required to substantiate this conclusion. According to the differences in phenotypes shown by different alleles, it can be suggested that *IBR5* may have multiple functions in plant growth and development, and some of which may be independent of IBR5.1 phosphatase catalytic activity.

The argument that IBR5.1 may have functions independent of its catalytic activity is reasonable, as there are many such known examples. For instance, the tyrosine phosphatase PTEN (phosphatase and tensin homolog deleted on chromosome 10) modulates p53 function independent of its catalytic activity in mice [31]. CTD phosphatase, a type-2C protein phosphatase, recycles RNA polymerase II by de-phosphorylating it. However, CTD phosphatase also promotes RNA elongation irrespective of its catalytic activity [32]. According to a recent study, different mutations that suppress *ibr5* auxin resistance restored distinct *ibr5* phenotypes [33]. Therefore, it is possible that IBR5 is involved in different signaling pathways.

### IBR5.1 uncouples Aux/IAA degradation from auxin-induced gene expression

The rate of degradation of *AXR3NT-GUS* reporter is enhanced in *ibr5-1*, while auxin induced *DR5-GU*S expression is diminished [6], [7]. Similar results were obtained with *ibr5-4*, which lacks phosphatase activity (Figure 3a, b). Therefore, it is likely that both auxin induced Aux/IAA degradation and gene expression are dependent on the catalytic activity of IBR5.1. In contrast to reduced *DR5::GFP* expression in *ibr5-4*, expression of *DR5::Venus* is greatly enhanced in the *IBR5.1-Myc* over-expression background (Figure 3c–e). These results suggest that the mechanism of auxin induced gene expression is a far more complex process than previously envisioned, and IBR5 acts as a negative regulator of Aux/IAA degradation, but functions as a positive regulator of auxin-induced gene expression, essentially decoupling these two processes. It is possible that IBR5.1 acts to fine tune both Aux/IAA degradation and auxin-induced gene transcription, presumably by regulating the phosphorylation status of protein/s involved in these two processes. However, to date the only known target for IBR5 is MPK12 [9], and how MPK12 regulates auxin signaling is not yet clear.

### The expression pattern of IBR5 correlates with its functions

The expression pattern of the translational reporter *IBR5::IBR5.1-GUS* was more restrictive than the transcriptional reporter construct described in [6]. Interestingly, the expression pattern of the IBR5.1 translational fusion was similar to the expression patterns of translational GUS fusions of TIR1/AFB auxin co-receptors [34]. IBR5.1 expression in cotyledons, shoot vasculature and lateral root initiation sites correlated with *ibr5* mutant phenotypes. It is also important to notice the absence of *IBR5* expression in dividing cells of the primary root tip and lateral root primordia (Figure 6d,g,i,j). Nevertheless, IBR5.1 expression is apparent in surrounding cells that are not actively dividing, suggesting that IBR5 may act as a negative regulator of cell division.

### IBR5 is involved in salinity, osmotic and oxidative stress responses

The *Arabidopsis* dual specificity phosphatase, MKP1, is involved in UV radiation and salinity stress tolerance [35]. MKP2, another dual specificity phosphatase, responds to oxidative stress [36]. Primary root elongation of *ibr5-1* is resistant to the stress hormone ABA [6]. MPK12, the only known target of IBR5, mediates biotic stress tolerance via ROS [14]. According to our physiological data, all three *ibr5* mutant alleles are resistant to salinity and osmotic stress. These observations correlate with the recent finding that over-expression of *OsIBR5* in tobacco confers sensitivity to drought [15]. However, the expression of *IBR5* is down-regulated by the above stresses and ABA. Stress conditions are known to induce ABA levels [37]. Thus it is possible that salinity and osmotic stresses induce ABA levels that will then down regulate *IBR5* expression. Conversely, *ibr5-1* and *ibr5-5* are more sensitive (Figure 7g) to methyl viologen (MV). MV induces oxidative stress [38]. This suggests that IBR5 is also involved in oxidative stress tolerance.

### IBR5 may represent a missing link of the ABP1 - SCF^TIR1/AFBs^ pathway

A recent study indicates that ABP1 is a negative regulator of the SCF^TIR1/AFBs^ pathway. Similar to *ibr5-1* and *ibr5-4* mutants, *AXR3NT-GUS* degradation is accelerated in ABP1 conditional mutant [8]. The authors show that the accelerated degradation of *AXR3NT-GUS* is not due to increased levels of auxin in this conditional mutant. *ibr5* mutants also do not exhibit any phenotypes indicative of IAA overproduction. Lack of IBR5.1 phosphatase activity paradoxically uncouples Aux/IAA degradation from auxin induced transcription (Figure 3b). Intriguingly, a similar effect is observed in ABP1 conditional mutant. Although, the loss of ABP1 function enhances AXR3NT-GUS degradation [8], it does not affect auxin sensitive gene expression [39]. While ABP1 functions genetically upstream of TIR1/AFBs [8], IBR5 has been suggested to act downstream of TIR1/AFBs auxin receptors [7]. However, accumulating data suggest that IBR5 may act both upstream and downstream of TIR1/AFBs to fine regulate the auxin-induced gene expression. Previous studies have also shown that interdigitation of Arabidopsis pavement cells is dependent on ABP1 and Rho GTPase based auxin signaling [19]. We have shown that interdigitation of epidermal cells is defective in *ibr5-1* and *ibr5-5*, even though this phenotype is less apparent in *ibr5-4* (Figure 4f) or *35S::IBR5^C129S^* (Figure S8). It is possible that the residual phosphatase activity of *ibr5-4* (Figure 2b) is sufficient to overcome a drastic effect on cellular interdigitation. Alternatively, this phenotype may not be dependent on phosphatase activity. Taken together, our results along with previous work suggest that *IBR5* may be the missing link between ABP1 and SCF^TIR1/AFBs^ dependent pathways. However, further work is necessary to support this notion.

In summary, we describe two new *ibr5* mutant alleles that give new insight into the functions of *IBR5* in auxin and ABA responses. We clearly show that *IBR5* is alternatively spliced to generate two isoforms, IBR5.1 and IBR5.3 that may have distinct and overlapping functions in growth and development. While IBR5.1 shows phosphatase activity, this catalytic activity may not be necessary for its full spectrum of functions. Comparison of mechanisms by which IBR5 and ABP1 regulate auxin signaling, it can be speculated that IBR5 and ABP1 share a common pathway to negatively regulate SCF^TIR1/AFBs^ dependent auxin signaling.

## Materials and Methods

### Plant Growth Conditions

*Arabidopsis thaliana* (L.) Heynh. Var. *Columbia* (Col-0) was used as the wild type. All mutant lines used in this study were in Col-0 background. *ibr5-1* and *35S::IBR5^C129S^* seeds were kindly provided by Dr. Bonnie Bartel, Rice University. Seeds were surface sterilized with 40% bleach with 0.1% TritonX-100 and excessively rinsed with sterile distilled water. Seeds were plated on *Arabidopsis thaliana* medium with 1% sucrose (ATS), pH 5.6. The plates were incubated at 4°C for 24 hours and then transferred to a growth chamber at 22°C with continuous illumination [40]. All experiments on sterile media were performed in the same growth chamber. Experiments with potted plants were carried out in a plant growth room at 25°C under continuous illumination.

### Transgenic constructs

To prepare *35S:*:*IBR5.1-Myc* and *35S*::*ibr5-4-Myc* constructs, the coding region was amplified from wild type and *ibr5-4* cDNA respectively using IBR5 BamH1 F/IBR5 Sal1 R primers (Table 1). Amplified DNA fragments were cloned into modified pBluescript vector containing 9× Myc tag sequence. Coding sequences along with Myc tag sequences were released from pBluescript plasmid using *BamH*I and *Kpn* I restriction digestion. Digested products were cloned into pROKII binary vector. *35S::IBR5.3*-*Myc* construct was prepared in the same manner by using IBR5 BamH1 F/IBR5-5 Myc Sal1 R primers (Table 1). To generate *IBR5::IBR5-GFP* construct, *IBR5* gene (2000 bp upstream of ATG was selected as promoter) was amplified using pIBR5 HindIII F/IBR5 Sal1 R primers (Table 1) and cloned into pBluescript vector, and subsequently into the modified pBI101.1-GFP vector. To generate the *35S::IBR5.1-GFP* and *35S::IBR5.3-GFP* constructs, DNA fragments were amplified using IBR5 pENTR F/IBR5 Sal1 R and IBR5 pENTR F/IBR5 Spl pENTR R primers (Table 1) respectively, and were cloned into pENTR vector. Subsequently DNA fragments were cloned into pB7FwG2.0 gateway binary vector by LR clonase reaction according to manufacturer's instructions (Invitrogen). Phusion DNA polymerase (NEB) was used in all DNA amplifications. These constructs were shuttled into *Agrobacterium tumefaciens* strain GV3101 and subsequently transformed into Arabidopsis plants for stable expression [41]. Homozygous lines expressing transgenes were selected using antibiotic resistance, and the expressions were confirmed by western blot analysis using anti-Myc antibody(Covance). Transient expression in *Nicotiana benthamiana* leaves was carried out as described previously [42].

**Table 1:** Primers used in the study.

Name	Sequence
IBR5 BamH1 F	5′ TTGGATCCCAAATTATGAGGAAGAGAGAA 3′
IBR5 Sal1 R	5′ CTGGTCGACAGAGCCATCCATTGCAATATC 3′
IBR5 pENTR F	5′- CACC CAAATTATGAGGAAGAGAGAAAGAG 3′
pIBR5 HindIII F	5′ CACCGAAGCTTTCAGATTTGATCCGGTGAG 3′
IBR5 Spl XbaI R	5′ GCTCTAGATCCTGCAGTTGTTGGTA 3′
IBR5-5 Myc Sal1 R	5′ CCGTCGACAA CTCCTGCAGT TGTTG 3′
IBR5 1F	5′ AAGGGTTTTCTCTGATCTGGGT 3′
IBR5 2F	5′ TGAGAAGGACAAGGCACGTGT 3′
IBR5 R	5′ CTAAGAGCCATCCATTGCAATATC 3′
ibr5-1 dcap F	5′ TCCTCCGTCTGTGAAATCAAG 3′
ibr5-1 dcap pst1 R	5′ GGAAAAGCACTGACGTGGACCTGCA 3′
ibr5-4 dcap F	5′ TCGGTAGTTACGACAACGCTTCTC 3′
ibr5-4 dcap R	5′ ACAACAACCGCTGGTGATCTACTGATA 3′
UBA F	5′ AGTGGAGAGGCTGCAGAAGA 3′
UBA R	5′CTCGGGTAGCACGAGCTTTA 3′
qIBR5 F	5′ TAGATCACCAGCGGTTGTTGTAGC 3′
qIBR5 R	5′TGTCAGTGCTTGGTCTCCGTTG 3′

### Immuno-precipitation(IP) of Myc tagged IBR5 and ibr5-4

Seedlings carrying *IBR5-Myc*, *ibr5-4*-Myc and Col-0 were grown for 10 days as described above. Total protein was isolated from 10 day old seedlings using IP extraction buffer (10% Glycerol, 25 mM Tris pH 7.5, 1 mM EDTA, 150 mM NaCl, 10 mM DTT, Protease inhibitor cocktail). 1 mg of total protein from *IBR5-Myc*, *ibr5-4-Myc* and Col-0 were incubated with anti-Myc antibody conjugated agarose beads (Clontech) for 3 hrs at 4°C. Immuno-precipitate was washed 5 times, each 5 minutes with IP washing buffer (IP extraction buffer +0.5% tween 20). 10% of the immuno-precipitates were separated on SDS PAGE gel and transferred on to a PVDF membrane. Proteins were visualized by western blot analysis using anti-Myc antibody (Covance).

### OMFP phosphatase assay

Phosphatase activity of IBR5 was quantified as previously described [9]. The assay was carried out at 25°C. Equal amounts of immuno-precipitated *IBR5-Myc* and *ibr5-4*-Myc proteins were incubated in 100 ul of phosphatase buffer containing 50 mM TRIS–HCl (pH 8), 150 mM NaCl, 1 mM EDTA and 500 µM 3-O-methylfluorescein Phosphate (OMFP). 2 ul samples were taken out at each time point and absorbance was measured at 477 nm using nano-drop spectrophotometer (Thermo scientific, ND-1000).

### Phenotypic characterization

All experiments were repeated at least three times. For primary root elongation assays with auxin and ABA, seedlings were first grown for four days in ATS media [43] and transferred to media containing different concentrations of auxin or ABA. Seedlings were grown for four additional days and primary root length was measured. For germination and cotyledon greening assays, seedlings were grown for seven days on ATS media containing indicated concentrations of NaCl, mannitol, ABA or MV. The number of total seedlings and the seedlings with green cotyledons were counted, and the percentage of green cotyledons was calculated.

For lateral root counts, seedlings were grown on unsupplemented media for 12 days. Fully emerged lateral roots and emerging lateral root primordia were counted under a dissecting microscope. Vascular patterning in 8 day-old cotyledons was observed after bleaching with acetone for 24 hrs. For adult plant phenotypes, wild type and mutant plants were grown on soil for 6 weeks, and plant height, rosette leaves and cauline leaves were compared. For seed size comparison, dried seeds from mature plants were collected and photographed. Length of the seeds was measured using ImageJ software [44].

### Histochemical staining and Quantitative β-glucuronidase assay

Histochemical staining of seedlings for GUS assays and quantitative β-glucuronidase assays were carried out as previously described [45]. Fluorescence was measured at a wavelength of 460 nM using a luminometer (Turner, Sunnyvale, CA, Model number-9200-002). All the experiments were carried out in triplicate.

### Microscopy

Imaging was done using Olympus FV1000 confocal microscope and analyzed using Olympus fluoview software. For sub-cellular localization analysis, Arabidopsis seedlings harboring *IBR5::IBR5.1-GFP* were grown for four days. For transient expression, *Nicotiana benthamina* leaves were injected with *Agrobacterium tumefaciens* carrying *35S::IBR5.1-GFP* and *35S::IBR5.3-GFP*, and leaf epidermal cells, were observed two days after the transfection. GFP was imaged and analyzed along with DAPI (Excitation-488 nm and 330 nm respectively, 40× oil immersion, NA 1.3). For imaging epidermal cells, seedlings were grown for seven days on unsupplemented media and stained with propidium iodide. Lower epidermis of stained cotyledons was imaged and analyzed (excitation, 555 nm; emission, 570–610 nm, 20× water immersion, NA 0.95).

### HS::AXR3NT-GUS, DR5::GFP and DR5::Venus analysis

*ibr5-4* was crossed into the *HS::AXR3NT-GUS* line and homozygous lines for both *ibr5-4* and *HS::AXR3NT-GUS* were selected by PCR using *ibr5-4* decap primers (Table 1). Homozygous seedlings were grown for four days on unsupplemented media and heat shocked for two hours at 37°C. Seedlings were then transferred to room temperature and fixed after indicated time intervals. Fixed seedlings were stained as described in [45]. *ibr5-4* was crossed into a line carrying *DR5::GFP* in wild type. Seedlings homozygous for both *ibr5-4* and *DR5::GFP* were grown for four days on unsupplemented media and transferred into media containing 85 nM 2,4D or 10 µM picloram for 12 hrs. GFP was imaged as described above. Gain and dynamic range settings were calibrated on control GFP expressing roots and then kept unchanged for recording of images of the roots with various treatments.

*35S::IBR5.1-Myc* line was crossed with the *DR5::Venus* reporter line. Seedlings homozygous for both *IBR5.1-Myc* and *DR5::Venus* were grown for four days on unsupplemented media and Venus was imaged as described above. For Venus quantification, image series were separated into red, green, and blue channels and background-corrected. Foci with GFP Expression above threshold levels were automatically counted using the analyze particles function in ImageJ [44].

### RNA preparation and qRT-PCR analysis

Wild type seedlings were grown for 4 days in ATS media and treated with NaCl, mannitol or ABA for 18 hours. Seedlings were then frozen in liquid nitrogen. Total RNA was extracted using TriReagent (Sigma) according to the manufacturer's instructions. RNase-free DNase was used to remove any contaminating DNA from the extract. cDNA was synthesized using total RNA and Superscript reverse transcriptase (Invitrogen) following the manufacturer's instructions. Following PCR program was used with specific primers as presented in Table 1; 55°C for 10 min, 95°C for 2 min, 40 cycles of 95°C for 15 s and 60 for 1 min. Primer efficiencies and relative expression levels were calculated using the comparative C_T_ method (User Bulletin 2, ABI Prism 7700 Sequence Detection System). 2^−ΔΔC^_T_ values of control samples were normalized to 1.

### Data analysis

For statistical comparison of data, single factor ANOVA and Tukey's HSD test were performed using “R” software (version 2.13.2, The R Foundation for Statistical Computing, ISBN 3-900051-07-0)

## Supporting Information

Figure S1 **Protein sequence alignment of IBR5.1, IBR5.3 and the predicted** ***ibr5-5*** **unspliced peptide sequence.** *ibr5-5* unspliced peptide sequence was predicted using ExPASy translate tool (http://web.expasy.org/translate/). IBR5.1 and IBR5.3 protein sequences (http://www.arabidopsis.org) were aligned with the predicted *ibr5-5* unspliced peptide sequence using the T-coffee multiple alignment tool (http://tcoffee.vital-it.ch/apps/tcoffee/index.html). The conserved phosphatase catalytic domain is underlined. The portion underlined in red indicates the highly conserved catalytic site.
(TIF)

Figure S2 **Catalytic activity of IBR5.3-Myc protein.** a) Total protein was isolated from transgenic plants over-expressing IBR5.3-Myc. The tagged protein was immuno-precipitated using anti-Myc antibody. 10% of the immuno-precipitate was visualized by western blotting using anti-Myc antibody. b) Phosphatase activity of IBR5.3-Myc was measured using OMFP assay. Reactions were carried out in triplicate. Error bars indicate standard deviations from the mean.
(TIF)

Figure S3 **Vascular patterning of** ***ibr5*** **alleles.** Seedlings were grown for 8days on unsupplemented media and cotyledons were bleached in acetone for 24 hrs prior to photographing with bright field microscopy (Nikon SMZ1500).
(TIF)

Figure S4 **Hypocotyl length of** ***ibr5*** **alleles.** a) Seedlings were grown for 5days on unsupplemented media and photographed using bright field microscopy (Nikon SMZ1500). Hypocotyl length was measured using ImageJ software. b) Primary root length of *ibr5* alleles. Seedlings were grown for 4days on unsupplemented media, and root length was measured. Error bars indicate standard deviations from the mean. Stars indicate that the means differ significantly from the control; letters indicate the samples that differ significantly from each other (n = 15, ANOVA, Tukey's HSD, P<0.05).
(TIF)

Figure S5 **Inhibition of primary root elongation by auxin.** Seedlings were grown for four days on unsupplemented media and transferred on to media containing 85 nM 2,4D (a) or 10 µM IBA (b). Seedlings were grown for four additional days and primary root length was measured. Results were standardized against unsupplemented media. Error bars indicate standard error of the mean. Stars indicate that the means differ significantly from the control. Letters indicate the samples that differ significantly from each other (n = 15, ANOVA, Tukey's HSD, P<0.05).
(TIF)

Figure S6 **Sub-cellular localization of IBR5.1 and IBR5.3.** *35S::IBR5.1-GFP* (a) and *35S::IBR5.3-GFP* (b) reporter constructs were transiently expressed in *Nicotiana benthamiana* leaves. Epidermal cells were imaged two days post-transfection, using Olympus FV1000 confocal microscopy. Nuclei were visualized using DAPI nuclear stain. Images were analyzed using Olympus fluoview software.
(TIF)

Figure S7 ***IBR5::IBR5.1-GUS*** **expression in dark grown seedlings.** *IBR5::IBR5.1-GUS* translational reporter construct was used to examine tissue specific expression of IBR5.1. Four day old dark grown seedlings carrying *IBR5::IBR5.1-GUS* were fixed and stained for GUS. Images were acquired using bright field microscopy (Nikon SMZ1500).
(TIF)

Figure S8 **Complementation of defective interdigitation of epidermal cells in** ***ibr5*** **mutants by IBR5.1-Myc.** IBR5.1-Myc and IBR5.3-Myc were overexpressed in different *ibr5* mutant alleles using 35SCaMV promoter. Propidium iodide stained lower epidermis of seven-day old cotyledons were imaged using Olympus FV1000 confocal microscopy. The uppermost panel indicates the images of the lower epidermis of Col-0 and *ibr5* mutants. The lower epidermis of *35S::IBR5^C129S^* was also included for comparison.
(TIFF)

Figure S9 **Inhibition of primary root elongation by NaCl and Mannitol.** Seedlings were grown for four days on unsupplemented media and transferred on to media containing 100 mM NaCl or 100 mM mannitol. Seedlings were grown for four additional days, and primary root length was measured. Results were standardized against unsupplemented media. Error bars indicate standard error of the mean. Stars indicate that the means differ significantly from the control (n = 15, ANOVA, P<0.05).
(TIFF)
