# Supplementary figures and images for: Alternative Splicing of Arabidopsis IBR5 Pre-mRNA Generates Two IBR5 Isoforms with Distinct and Overlapping Functions
Source: PLoS One. 2014 Aug 21;9(8):e102301. doi: 10.1371/journal.pone.0102301 (PMC4140696; doi:10.1371/journal.pone.0102301)

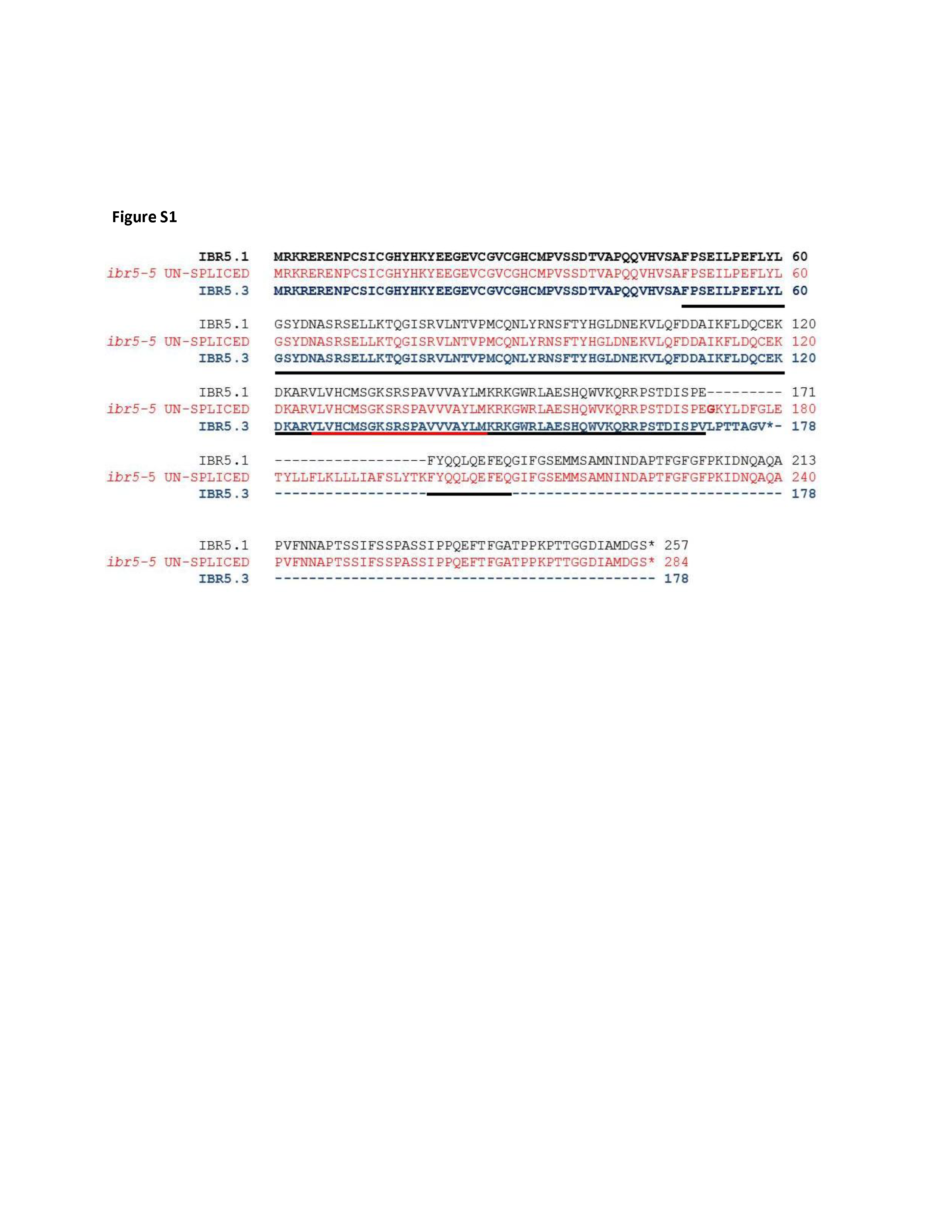

Supplement: Figure S1 — Protein sequence alignment of IBR5.1, IBR5.3 and the predicted ibr5-5 unspliced peptide sequence. ibr5-5 unspliced peptide sequence was predicted using ExPASy translate tool (http://web.expasy.org/translate/). IBR5.1 and IBR5.3 protein sequences (http://www.arabidopsis.org) were aligned with the predicted ibr5-5 unspliced peptide sequence using the T-coffee multiple alignment tool (http://tcoffee.vital-it.ch/apps/tcoffee/index.html). The conserved phosphatase catalytic domain is underlined. The portion underlined in red indicates the highly conserved catalytic site. (TIF) [file pone.0102301.s001.tif]

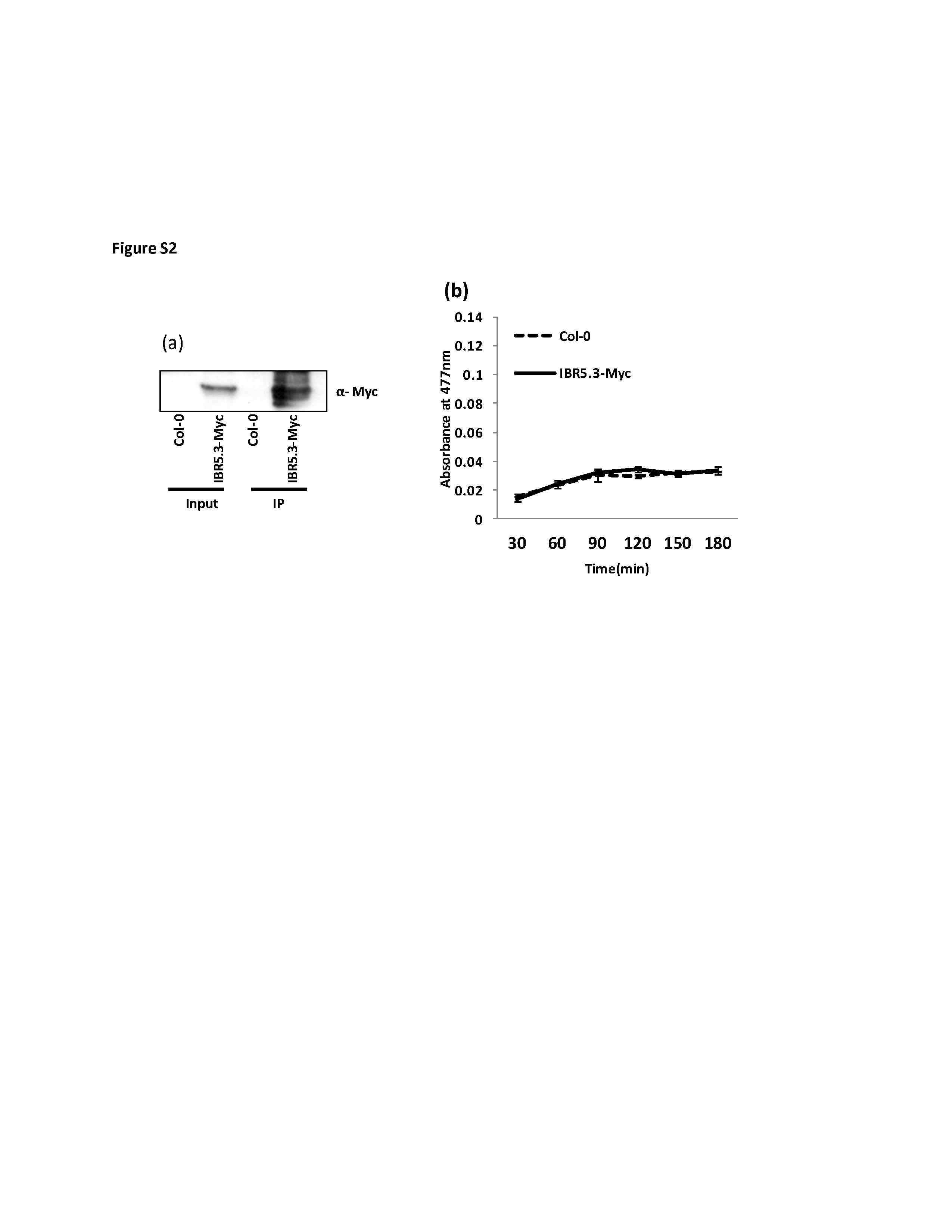

Supplement: Figure S2 — Catalytic activity of IBR5.3-Myc protein. a) Total protein was isolated from transgenic plants over-expressing IBR5.3-Myc. The tagged protein was immuno-precipitated using anti-Myc antibody. 10% of the immuno-precipitate was visualized by western blotting using anti-Myc antibody. b) Phosphatase activity of IBR5.3-Myc was measured using OMFP assay. Reactions were carried out in triplicate. Error bars indicate standard deviations from the mean. (TIF) [file pone.0102301.s002.tif]

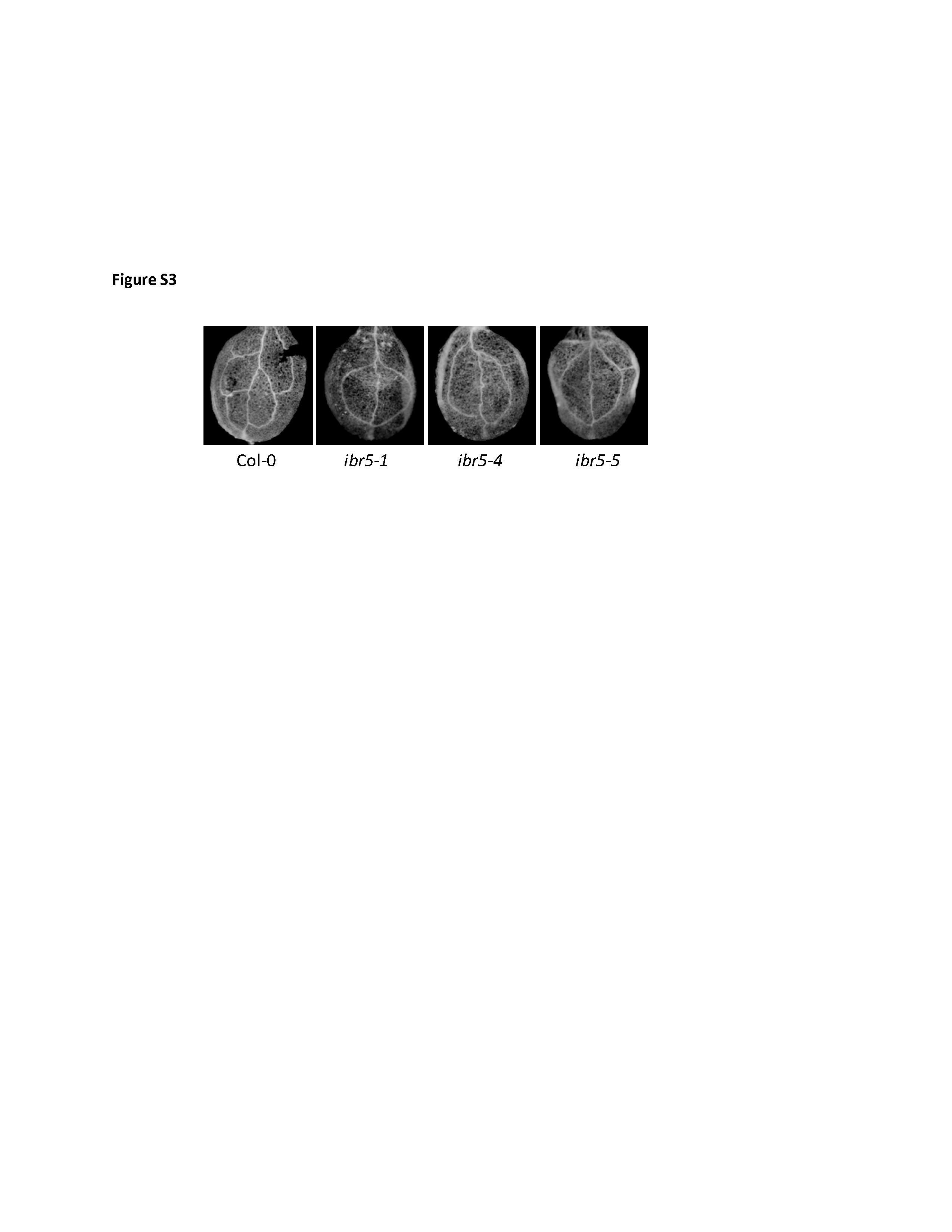

Supplement: Figure S3 — Vascular patterning of ibr5 alleles. Seedlings were grown for 8days on unsupplemented media and cotyledons were bleached in acetone for 24 hrs prior to photographing with bright field microscopy (Nikon SMZ1500). (TIF) [file pone.0102301.s003.tif]

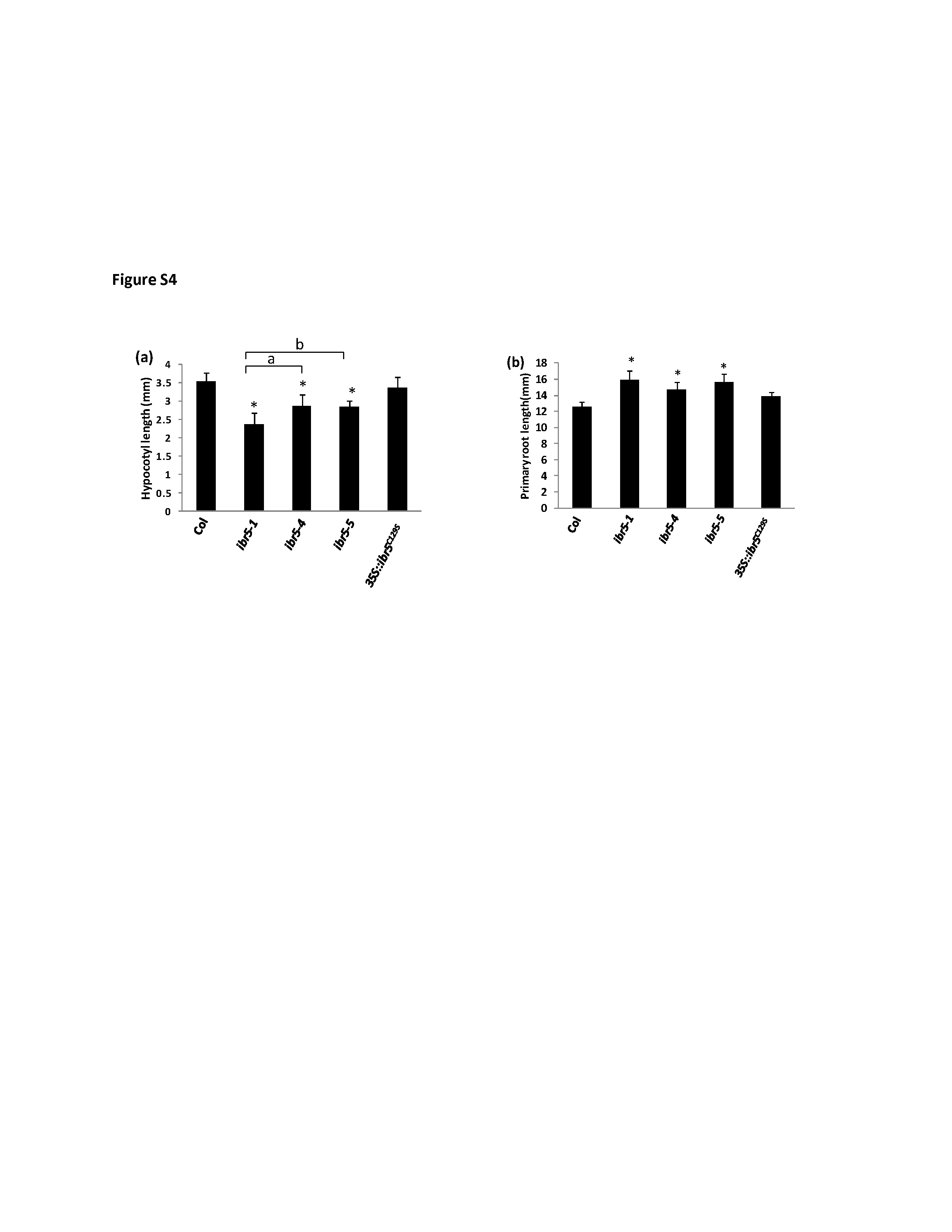

Supplement: Figure S4 — Hypocotyl length of ibr5 alleles. a) Seedlings were grown for 5days on unsupplemented media and photographed using bright field microscopy (Nikon SMZ1500). Hypocotyl length was measured using ImageJ software. b) Primary root length of ibr5 alleles. Seedlings were grown for 4days on unsupplemented media, and root length was measured. Error bars indicate standard deviations from the mean. Stars indicate that the means differ significantly from the control; letters indicate the samples that differ significantly from each other (n = 15, ANOVA, Tukey's HSD, P<0.05). (TIF) [file pone.0102301.s004.tif]

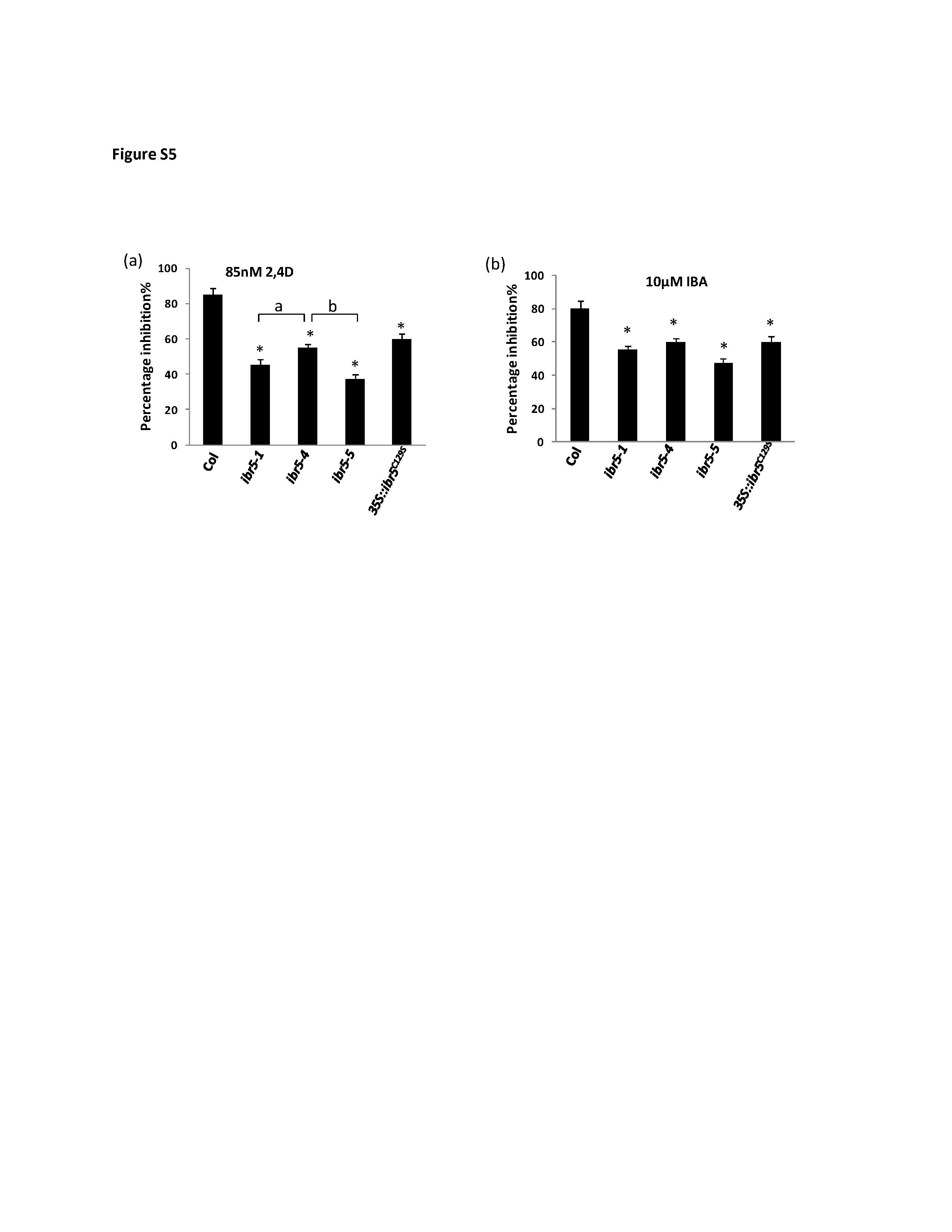

Supplement: Figure S5 — Inhibition of primary root elongation by auxin. Seedlings were grown for four days on unsupplemented media and transferred on to media containing 85 nM 2,4D (a) or 10 µM IBA (b). Seedlings were grown for four additional days and primary root length was measured. Results were standardized against unsupplemented media. Error bars indicate standard error of the mean. Stars indicate that the means differ significantly from the control. Letters indicate the samples that differ significantly from each other (n = 15, ANOVA, Tukey's HSD, P<0.05). (TIF) [file pone.0102301.s005.tif]

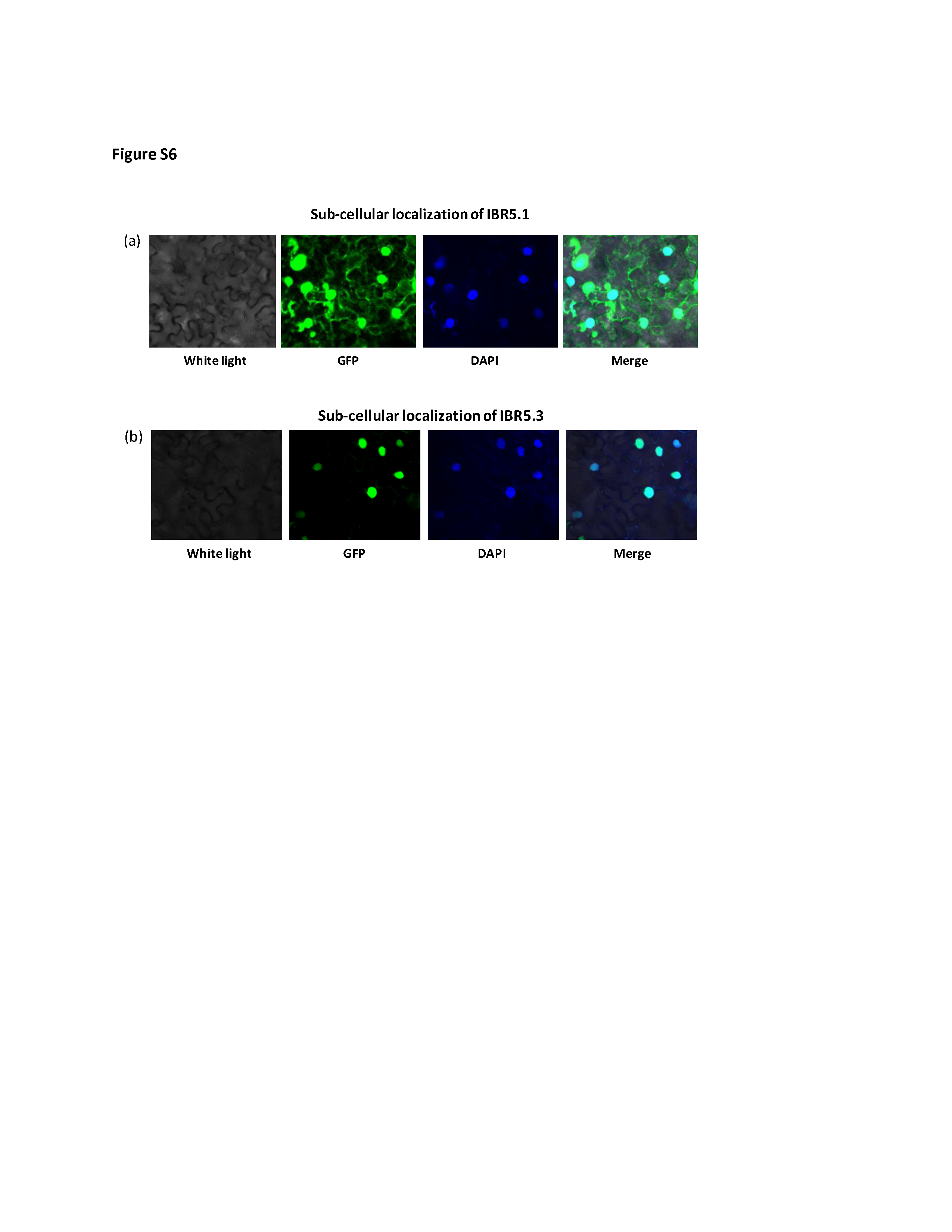

Supplement: Figure S6 — Sub-cellular localization of IBR5.1 and IBR5.3. 35S::IBR5.1-GFP (a) and 35S::IBR5.3-GFP (b) reporter constructs were transiently expressed in Nicotiana benthamiana leaves. Epidermal cells were imaged two days post-transfection, using Olympus FV1000 confocal microscopy. Nuclei were visualized using DAPI nuclear stain. Images were analyzed using Olympus fluoview software. (TIF) [file pone.0102301.s006.tif]

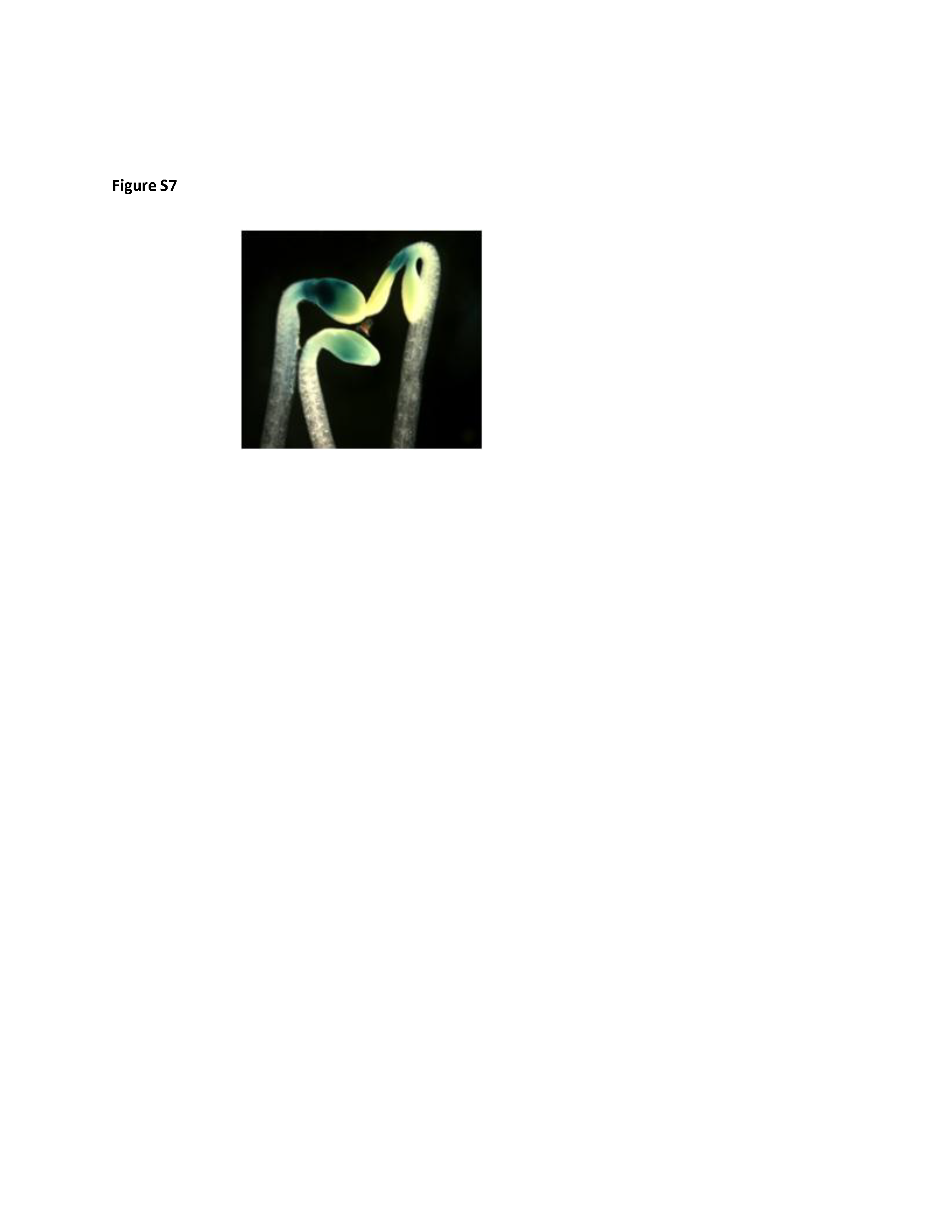

Supplement: Figure S7 — IBR5::IBR5.1-GUS expression in dark grown seedlings. IBR5::IBR5.1-GUS translational reporter construct was used to examine tissue specific expression of IBR5.1. Four day old dark grown seedlings carrying IBR5::IBR5.1-GUS were fixed and stained for GUS. Images were acquired using bright field microscopy (Nikon SMZ1500). (TIF) [file pone.0102301.s007.tif]

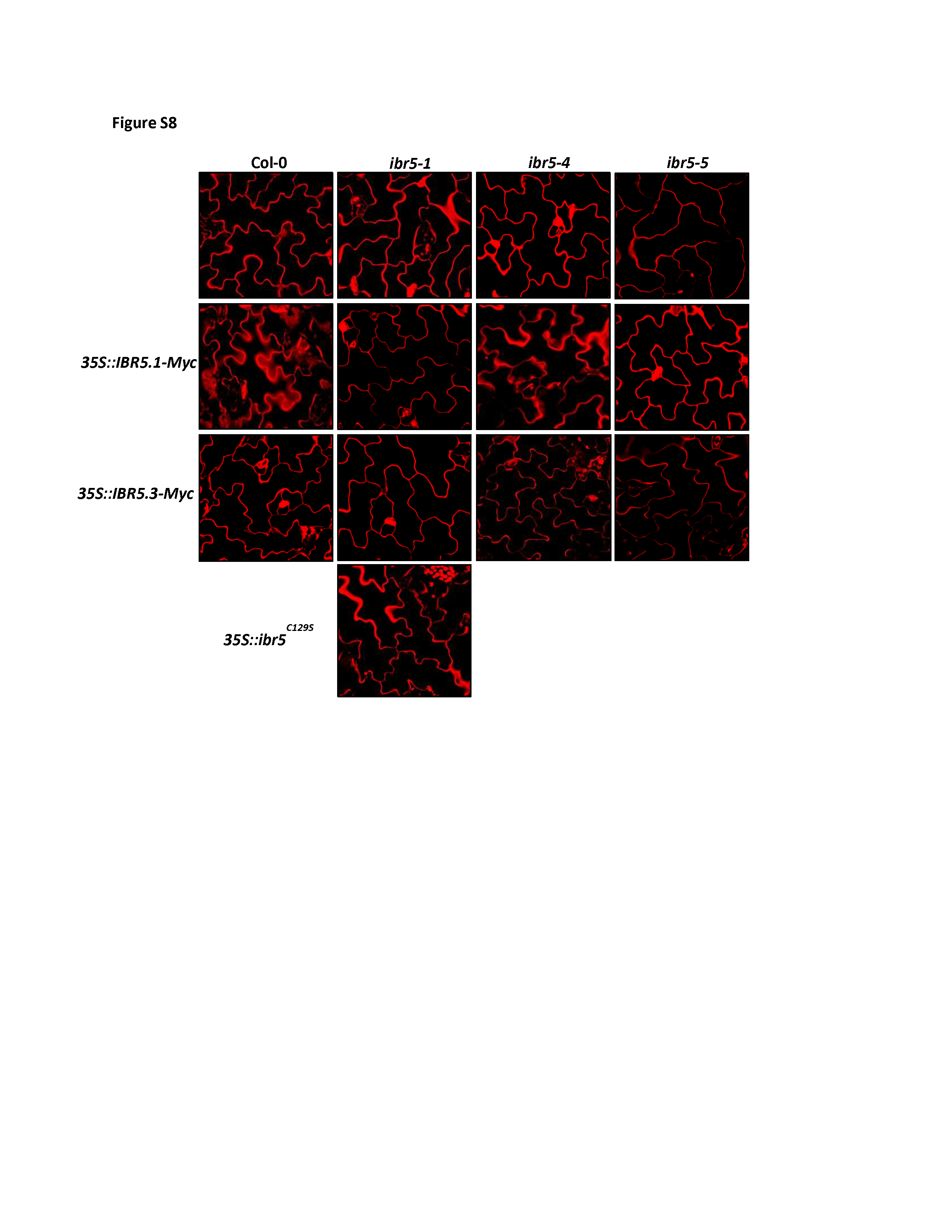

Supplement: Figure S8 — Complementation of defective interdigitation of epidermal cells in ibr5 mutants by IBR5.1-Myc. IBR5.1-Myc and IBR5.3-Myc were overexpressed in different ibr5 mutant alleles using 35SCaMV promoter. Propidium iodide stained lower epidermis of seven-day old cotyledons were imaged using Olympus FV1000 confocal microscopy. The uppermost panel indicates the images of the lower epidermis of Col-0 and ibr5 mutants. The lower epidermis of 35S::IBR5C129S was also included for comparison. (TIFF) [file pone.0102301.s008.tiff]

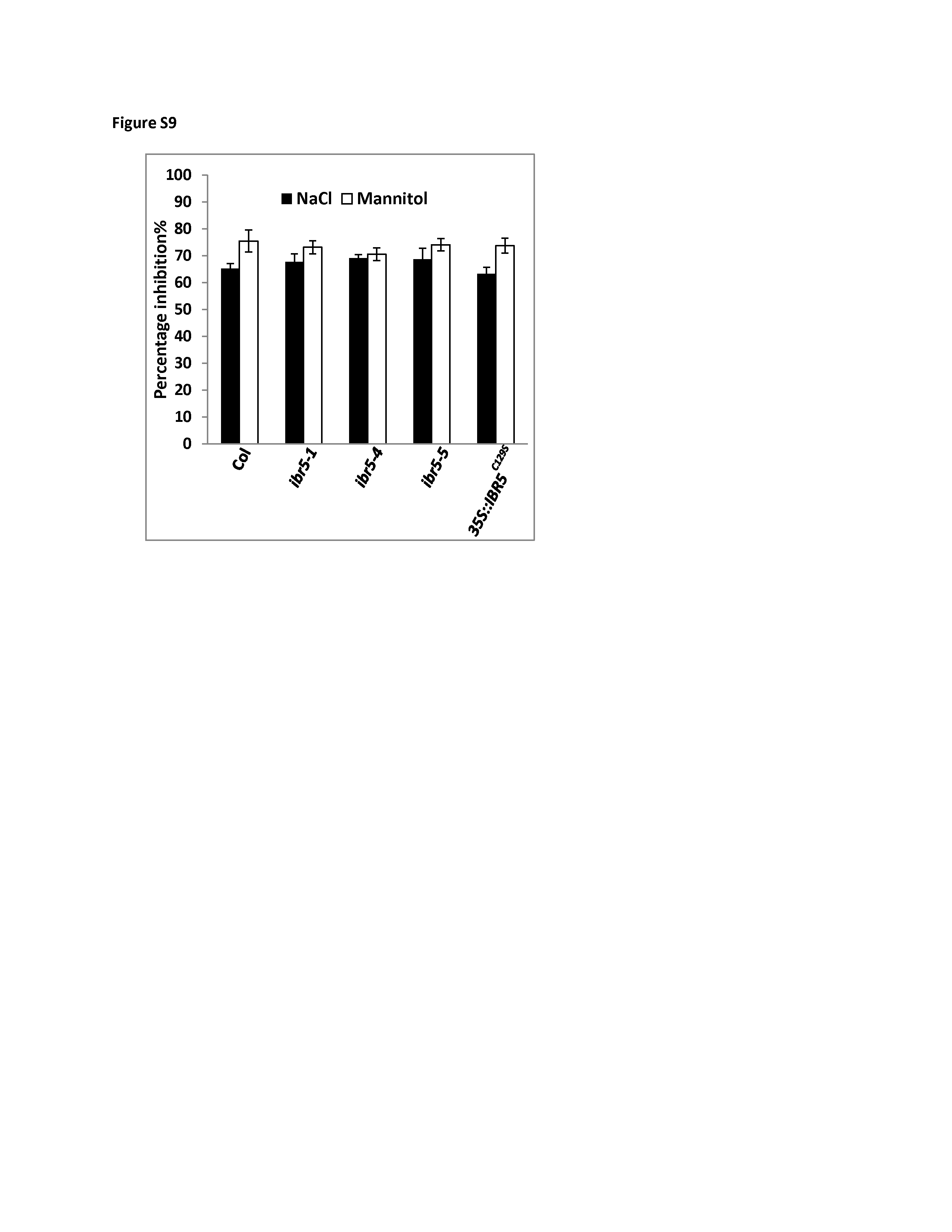

Supplement: Figure S9 — Inhibition of primary root elongation by NaCl and Mannitol. Seedlings were grown for four days on unsupplemented media and transferred on to media containing 100 mM NaCl or 100 mM mannitol. Seedlings were grown for four additional days, and primary root length was measured. Results were standardized against unsupplemented media. Error bars indicate standard error of the mean. Stars indicate that the means differ significantly from the control (n = 15, ANOVA, P<0.05). (TIFF) [file pone.0102301.s009.tiff]
